# Evaluating biocide efficacy in mixed-species biofilms: insights from a dual anaerobic biofilm reactor

**DOI:** 10.1038/s41529-025-00628-0

**Published:** 2025-07-31

**Authors:** Liam Jones, Niall Hanrahan, Maria Salta, Torben Lund Skovhus, Kathryn Thomas, Timothy Illson, Julian Wharton, Jeremy Webb

**Affiliations:** 1 School of Biological Sciences, University of Southampton, Southampton, UK; 2 School of Chemistry, Faculty of Engineering and Physical Sciences, University of Southampton, Southampton, UK; 3 Endures, MIC and Biofilm Department, Bevesierweg, Den Helder, The Netherlands; 4 Research Centre for Built Environment, Climate and Water Technology, VIA University College, Horsens, Denmark; 5 DNV, Holywell Park, Ashby Road, Loughborough, UK; 6 School of Engineering, University of Southampton, Southampton, UK; 7 National Biofilms Innovation Centre, Cardiff, UK

**Keywords:** Biochemistry, Microbiology, Environmental sciences, Chemistry, Materials science

## Abstract

Understanding biocide performance in mixed-species biofilms is critical to mitigating microbiologically influenced corrosion (MIC). In this study, a novel dual anaerobic biofilm reactor was used to evaluate glutaraldehyde efficacy under environmentally relevant conditions, using a complex microbial consortium from marine sediment. Despite biocide dosing, biofilms persisted and induced localized corrosion, indicating incomplete mitigation. Each biocide application led to an electronegative shift in *E*_corr_ and a reduction in ${{\rm{H}}}_{2}{\rm{S}}$ concentration, suggesting partial suppression of microbial activity. Raman spectroscopy and profilometry revealed differences in corrosion product composition and pit morphology between biotic and abiotic systems. 16S rRNA sequencing showed enrichment of stress-tolerant genera, including *Exiguobacterium* and *Serpentinicella*, consistent with increased chemical tolerance. These findings highlight the limitations of conventional biocide strategies and demonstrate the need for adaptive, community-informed treatment approaches. The dual-reactor model provides a robust platform for future MIC standardization efforts and mechanistic investigation of biofilm resilience under anoxic conditions.

## Introduction

To control the threat of microbiologically influenced corrosion (MIC), various mitigation strategies have been employed in the energy sector. These include chemical treatment with biocides^1–5^, corrosion inhibitors^6^, and surfactants^7^, as well as physical treatments using paints and coatings^8^, and mechanical cleaning/pigging. Additionally, adjustment of operational parameters, asset design alterations, and appropriate materials selection can also be employed to mitigate the threat of MIC. Among chemical treatments, corrosion inhibitors and biocides are the most widely used. Biocides are utilised for the inhibition of bacterial growth and excessive biofilm formation^3^. However, many traditional oxidising biocides are environmentally hazardous and may exert adverse effects on aquatic species due to the release of chemical byproducts^3^. Biocidal compounds, such as glutaraldehyde and THPS, which are non-oxidising control treatments, have been widely employed to mitigate MIC due to their broad-spectrum efficacy, biodegradability, safety, and cost effectiveness^9,10^. Yet, over time, sessile biofilm communities often develop increased tolerance to these chemical biocides^11^. Thus, there has been an increase in the development of novel eco-friendly biocidal compounds over recent years^12,13^. Understanding not only how these chemicals interact at the biofilm/metal interface where corrosion-promoting species drive MIC mechanisms, but also understanding how they are degraded, what chemical byproducts they produce, and how they subsequently interact with the environment is critical when trying to understand efficacy and improve sustainability.

Although traditional biocides can initially reduce microbial populations and slow the progression of MIC by disrupting biofilm formation and metabolic activity, their long-term effectiveness is often compromised by the development of biofilm-associated tolerance. This tolerance can be sustained over time, particularly in systems where repeated or sublethal biocide exposure selects for resilient subpopulations. Resilience arises from multiple factors, including the protective barrier formed by extracellular polymeric substances (EPS), spatial heterogeneity within the biofilm, and the metabolic versatility of mixed-species communities^14^. EPS can hinder biocide diffusion, creating concentration gradients that leave deeper biofilm layers less exposed. Within these layers, dormant persister cells and species capable of enzymatic detoxification may persist. Moreover, inter-species interactions, such as metabolic cooperation and quorum sensing, can further enhance community-level resistance^14^. In corrosion systems, biofilms also interact with corrosion products, which may bind or neutralize biocides, reducing their bioavailability^15,16^ While biofilm structures are often heterogeneous and can be highly complex and biomass-rich, these features vary depending on environmental conditions, community composition, and surface characteristics. Given that MIC is driven by the collective activity of diverse microorganisms, and that biofilm structure and function vary even within similar environments, understanding the spatial and functional dynamics of mixed-species biofilms is essential^12^. This complexity underscores the need for integrative testing platforms, such as the one employed in this study, that can evaluate biocide efficacy under realistic, environmentally relevant conditions.

The biofilm structure can affect the diffusion of antimicrobial compounds through the stratified biofilm layers, thereby reducing the concentration of antimicrobial compounds reaching the cells at the bottom of biofilms at the metal interface, which are often the corrosion-promoting species^1^. Additionally, the biofilm can also contribute to the formation of anaerobic microenvironments within the biofilm, which further supports the growth of strict anaerobes such as sulphate-reducing bacteria (SRB), key drivers of MIC. Therefore, cells at the interface of the metal surface will be exposed to sublethal concentrations of biocidal compounds and will have sufficient time to switch on the expression of antimicrobial-resistant factors and antimicrobial-degrading enzymes^1,17^. Biocides frequently fail to completely eradicate biofilm communities, leading to the persistence of microbial populations, as the energy sector commonly uses a cyclic biocide treatment protocol^18^. These residual biofilm communities can then regenerate when there is no biocidal treatment and continue to drive MIC mechanisms. Additionally, the efficacy of biocides can vary significantly depending not only on the starting composition of the mixed-species biofilm, but also on functional redundancy within the community and the spatial and metabolic heterogeneity of the biofilm itself. Microorganisms residing in the deeper layers of the biofilm often adopt a slow-growing or metabolically quiescent state, which renders them far more tolerant to antimicrobial agents than actively dividing planktonic cells typically used in standard MIC tests. This layered physiology, combined with overlapping metabolic capabilities across species, allows biofilms to maintain critical functions and resist clearance even when exposed to targeted biocides^14,15^. Additionally, different biocides have different modes of action. Thus, some microbial species within a mixed-species biofilm may be more resistant to biocidal treatments than others. There are diverse strategies which microorganisms can employ to enable survival. These include efflux pumps, enzymatic degradation, modification of membrane permeability, quorum sensing and horizontal gene transfer to name a few. Therefore, despite biocidal treatment, corrosion can persist due to the resilience and adaptability of microbial communities within mixed-species biofilms. These complexities further underscore the need for test systems that replicate the dynamic architecture and metabolic diversity of natural biofilms when evaluating biocide performance.

Despite the widespread application of biocides, relatively few studies evaluate their efficacy against mixed-species biofilms in long-term, flow-based systems that simulate environmental corrosion conditions, particularly under anoxic conditions relevant to MIC^1,3^. Much of the existing literature relies on short-term, static, or planktonic assays, which may not adequately capture the spatial and metabolic complexity of mature biofilms in industrial settings^2,19–21^. Sharma et al. demonstrated that glutaraldehyde alone was the most ineffective biocide tested against *D. ferrophilus* IS5, a model SRB known to utilise electrons directly from metal. Whereas benzalkonium chloride (BAC) was the most effective biocide in preventing biofilm formation and pitting on the carbon steel (CS) coupons^2^. However, they noted that MIC in the natural environment is due to the action of a mixed-species biofilm. And while pure microbial strains can provide insight to better understand MIC mechanisms, ultimately biocidal efficacy must be validated against mixed-species biofilms^2^ Salgar-Chaparro et al. stressed the importance of assessing mixed-species biofilms under different nutrient conditions to understand the effectiveness of biocide treatments^1^. Their results demonstrated that thicker biofilms, grown under continual nutrient replenishment, exhibited greater survival against a glutaraldehyde treatment compared to a biofilm grown under batch conditions without nutrient replenishment^1^. In this instance, it may be appropriate to enhance the biocide treatment with an adjunctive strategy. The efficacy of an adjunctive strategy using a combination of phenolic and quaternary ammonium compounds (QACs) has been reported against a mixed-species biofilm^3^. Chang et al. demonstrated a strong antimicrobial effect against a mixed-species biofilm isolated from the environment. The combination treatment demonstrated a synergistic effect to reduce bacterial growth compared to individual treatments^3^. These types of studies highlight the importance of evaluating the efficacy of different biocide treatments against mixed-species biofilms.

This study builds off a previous experiment^22^, in which the applicability of the novel dual bioreactor protocol was first investigated using a mixed-species biofilms under simulated marine conditions. This model enabled the simultaneous analysis of abiotic and biotic corrosion mechanisms under strictly anoxic conditions, using a complex mixed-species microbial consortium derived from marine littoral sediment. Importantly, we demonstrated that the predominant biofilm activity was driven by electroactive bacteria, including sulphate-reducing and iron-reducing species. Electrochemical measurements, along with 16S rRNA gene sequencing, confirmed the emergence of structurally complex biofilms capable of enhancing localized corrosion. This work provided a foundational framework for evaluating corrosion mechanisms in the presence of electroactive microbial communities and underscored the necessity of robust model systems that reflect the dynamic interplay between biofilms and metal substrates in natural environments. Importantly, that experiment did not examine biocide application using the model system. Here, we go one step further and propose a targeted approach to MIC mitigation by utilising the novel dual bioreactor protocol to evaluate and optimise biocidal treatment strategies. By utilising multiple lines of evidence (MLOE)^23^, the protocol incorporates a multi-disciplinary approach to gain a holistic understanding of biofilms, MIC, and biocidal efficacy. This research offers an innovative approach to refining biocidal application in a controlled environment, closely mimicking environmental conditions, and critically using a mixed-species biofilm. This approach aims to overcome the challenges of biofilm tolerance, offering a more sustainable and effective solution to mitigating the threat of MIC. It was hypothesized that over time, even with the application of biocide, the biotic condition would exhibit a greater incidence of pitting compared to the abiotic condition due to biofilm phenotype conferring an increased survival/recalcitrance.

## Results

### Visual observations

During the initial batch phase, a faint pink coloration from resazurin was observed in both reactors, indicating the presence of residual oxygen; this was due to the initial challenge of fully deoxygenating the 10 L media reservoirs and tubing during setup. After the onset of continuous flow, the abiotic reactor media shifted to an orange hue, while the biotic reactor developed a black-green coloration, consistent with the establishment of anoxic conditions and the accumulation of sulphide and biofilm-associated metabolites. Maintaining strict anoxic conditions at this scale proved operationally demanding, particularly during media changes and gas exchange, and may have contributed to transient redox fluctuations early in the experiment. Over the initial three-day batch phase, the abiotic media had no apparent visual changes, and the coupons maintained the silver-grey metallic lustre of the CS. It was not until the flow of fresh media was started on day 4 that any visual changes could be observed. The sterile abiotic reactor ASW media became reddish-brown in colouration with increased turbidity after the first week. Conversely, after inoculation of the biotic reactor ASW media, a black surface film was present on the steel coupons on day 1. At the end of the initial three-day batch phase, there was a low level of turbidity. After the flow of fresh ASW media was started on day 4, the ASW media was completely covered with black particulates and had a very high level of turbidity. On day 28, the biotic reactor was black/brown in appearance. Upon dismantling of the reactors on day 28 and retrieval of the coupon rods, there was a significant difference in the coupon appearances, see Supplementary Fig. 1. The abiotic surfaces were covered in a reddish-brown corrosion product. Whilst the biotic surfaces were generally also covered in a reddish-brown corrosion product, beneath which this was a black deposit strongly adhered to the metallic CS surface.

### Sulphide analysis

**Fig. 1:**
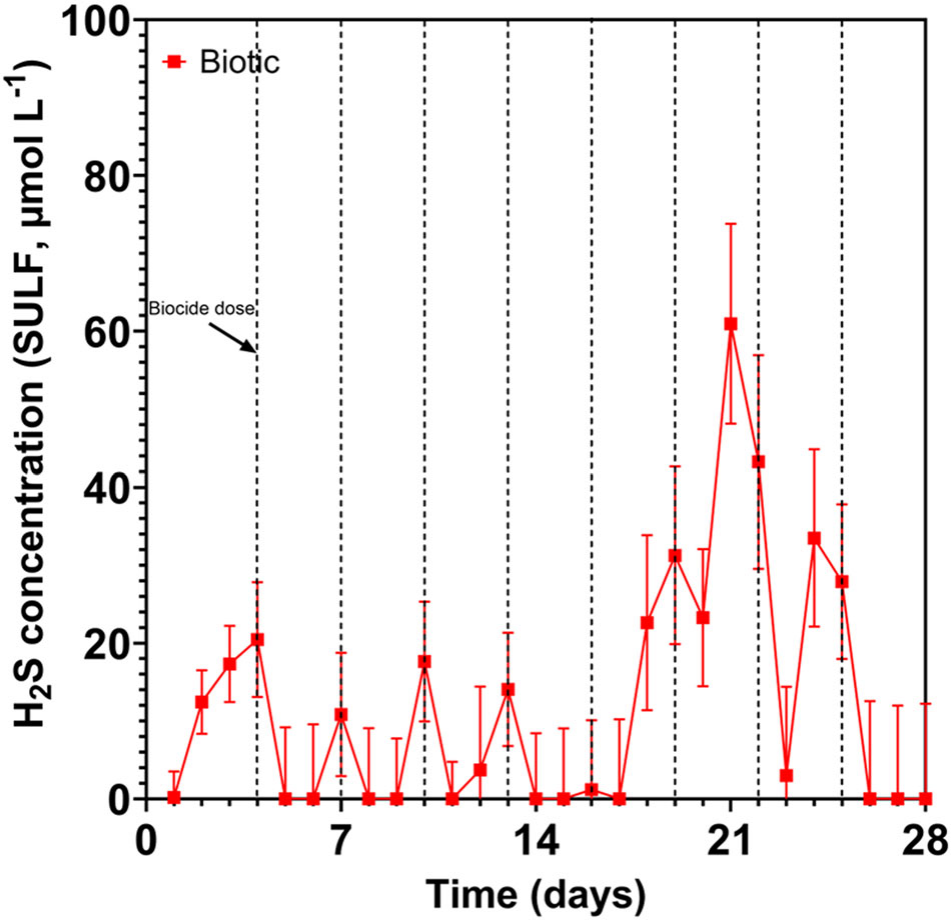
Hydrogen suphide concentrations over 28 days. Aqueous sulphide measurements (*SULF*, µmol L^–1^) for the biotic condition over 28 days (nb. measured the anaerobic nutrient-enriched artificial seawater media dosed bi-weekly with glutaraldehyde in situ adjacent to corroding UNS G10180 carbon steel). Dash vertical lines indicate biocide dosing time points.

Figure 1 shows the aqueous ${{\rm{H}}}_{2}{\rm{S}}$ concentrations monitored for the biotic anaerobic nutrient-enriched ASW media over the test duration. Unfortunately, whilst calibrating the microsensors prior to starting the experiment, the second microsensor was discovered to be damaged and not in working order. Consequently, only one microsensor was used for this study for the biotic condition and no data is available for the abiotic condition. For the biotic condition, the ${{\rm{H}}}_{2}{\rm{S}}$ concentration generally peaked prior to being dosed with glutaraldehyde, with a maximum concentration of 60 µmol L^–1^ measured on day 21. After each dose of biocide (every third day, starting on day 4), there was a decrease in the ${{\rm{H}}}_{2}{\rm{S}}$ concentration. Dissolved oxygen (DO) concentrations measured on day 28 were between 0.16 and 0.88 ppm in the 10 L media containers, 3.3 ppm in the abiotic and 0.2 ppm in the biotic reactor. The pH was not measured on completion of the experiment.

### Carbon steel surface analysis

**Fig. 2:**
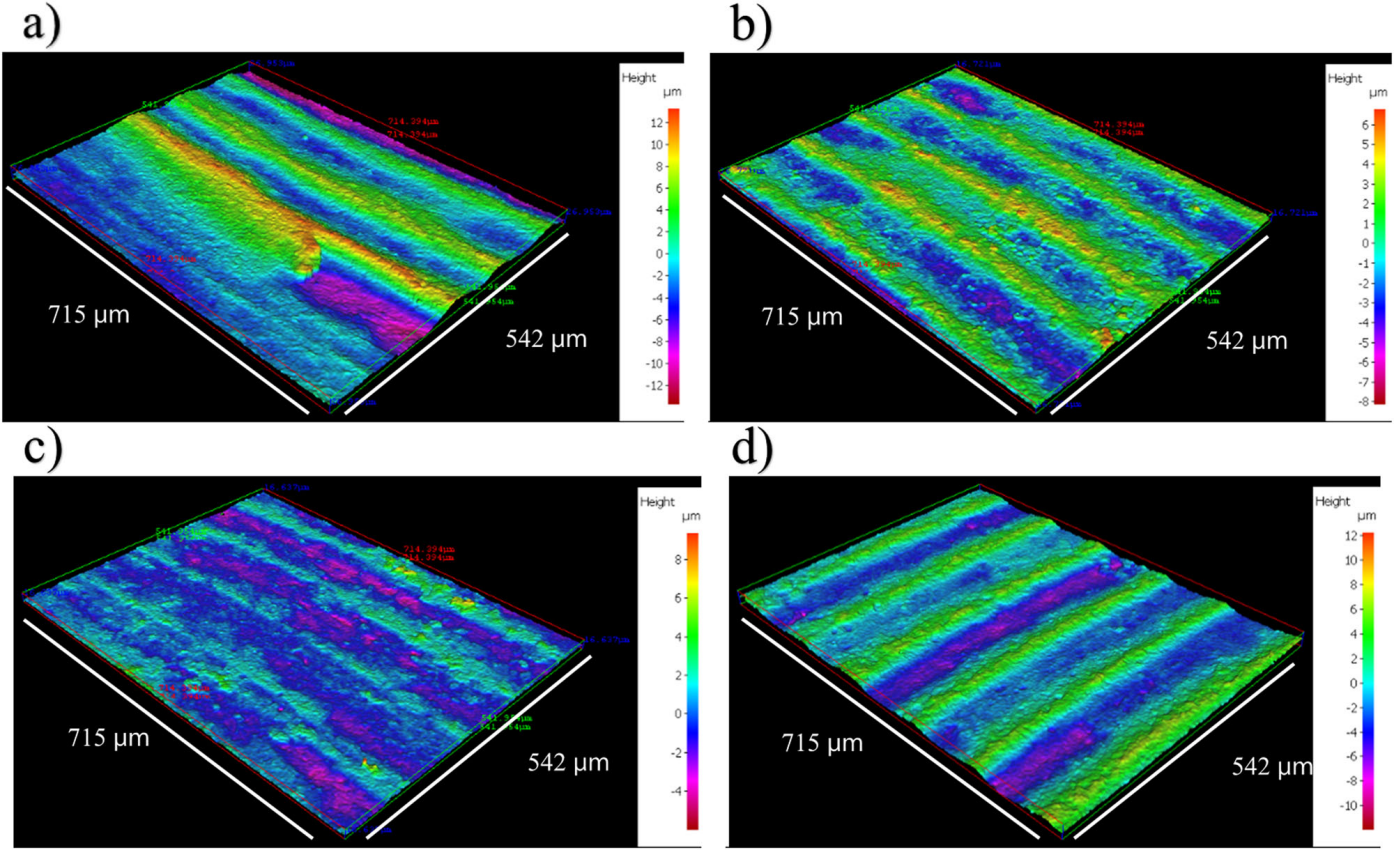
Three-dimensional optical surface profilometry of the cleaned UNS G10180 surfaces at day 28. AR coupons for: **a**, **b** abiotic and **c**, **d** biotic conditions, after exposure to anaerobic nutrient-enriched artificial seawater media dosed bi-weekly with glutaraldehyde for 28 days.

Supplementary Fig. 2 shows the CS surfaces on day 0. Supplementary Table 1 summarises the quantitative surface roughness profiles on both day 0 and day 28. Figure 2 shows the cleaned CS surfaces after 28 days, with biofilms and corrosion products removed to reveal the morphology of the surface degradation and to facilitate corrosion assessment. Surface profilometry revealed measurable changes in surface topography between day 0 and day 28 for both biotic and abiotic conditions. Prior to exposure, as-received carbon steel coupons displayed relatively low average surface roughness (*R*_a_ 1.2–1.3 µm), with uniform machining marks and minimal surface features. After 28 days, although the average roughness (*R*_a_) values remained relatively stable across both conditions, a modest reduction in parameters such as total height of the profile (*R*_t_), average maximum height of the profile (*R*_z_), and mean spacing of profile irregularities (*R*_sm_) was observed, indicating a smoothing of the overall surface profile likely due to generalized corrosion and loss of sharp features Notably, biotic coupons exposed to glutaraldehyde exhibited greater surface heterogeneity and localized pit features in post-exposure scans, consistent with biofilm-driven microenvironments that can promote localized corrosion despite biocidal treatment. In contrast, abiotic coupons demonstrated more uniform surface degradation. These data suggest that although glutaraldehyde dosing may have mitigated the extent of microbial activity, its effect was not sufficient to fully suppress biofilm-mediated corrosion processes over the exposure period. There were low levels of uniform or localised pitting corrosion present for both the abiotic and biotic condition. The abiotic average pit depths were 7.7 µm, with an average pit area of 2308 µm^2^ for any classified pits. The biotic average pit depths were 8.7 µm, with an average pit area of 8684 µm^2^ for any classified pits. However, only 2–3% of the analysed surfaces observed pitting. Again, for this study, a pit was classified as having a depth greater than 5 µm and an area greater than 650 µm^2^ ^24^.

**Fig. 3:**
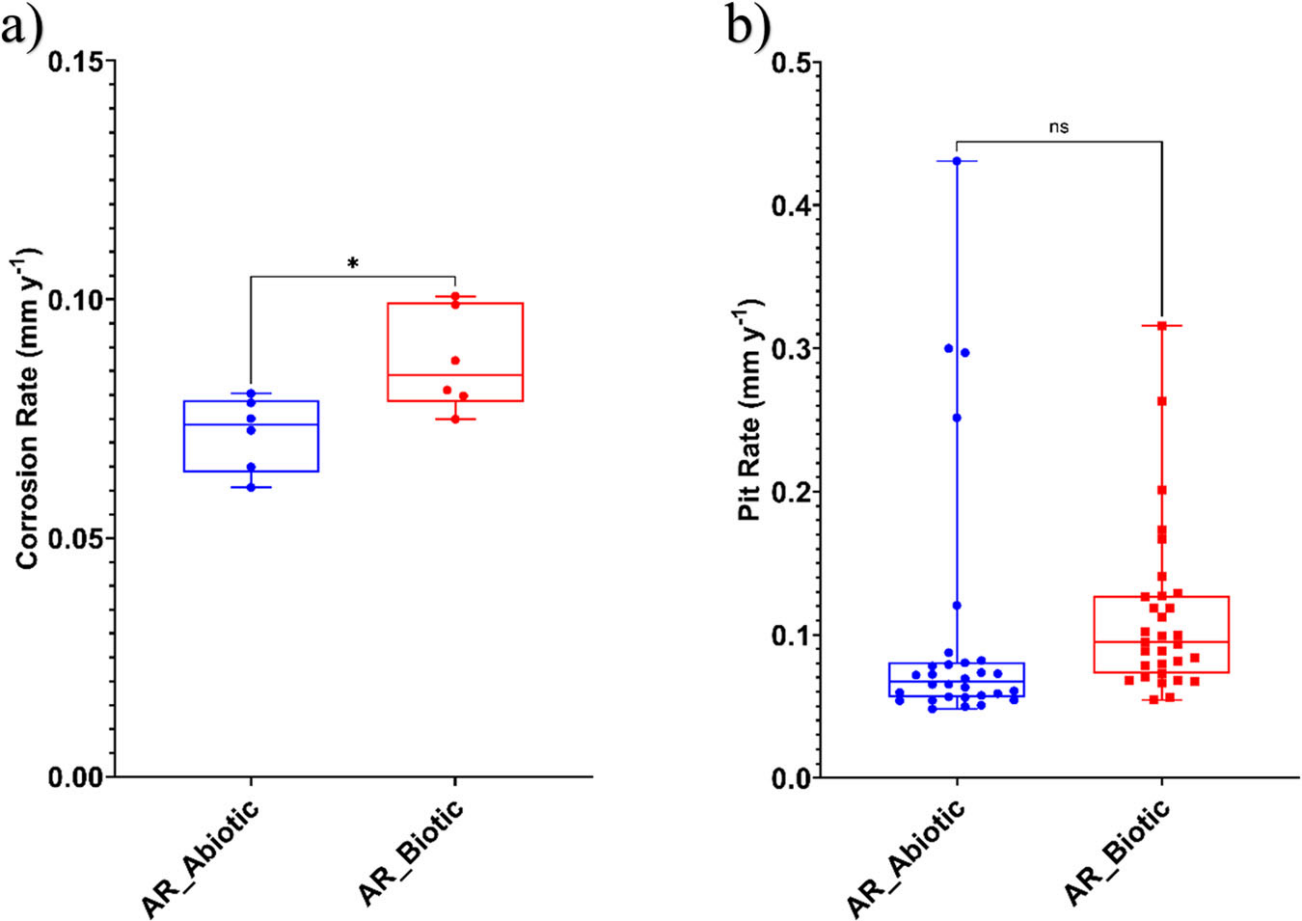
Abiotic and biotic corrosion performance after exposure to anaerobic nutrient-enriched artificial seawater media dosed bi-weekly with glutaraldehyde for 28 days. **a** corrosion rate via gravimetric analysis and surface profilometry assessed (*P* < 0.05) and **b** pit rate, for the AR coupons.

Figure 3a provides an evaluation of the CS corrosion performance after 28 days. For the abiotic condition, there was a significantly lower *CR* when compared to the biotic condition. According to the NACE SP0775-2023 assessment criteria, there was a moderate *CR* (between 0.025 and 0.12 mm y^–1^) in both the abiotic and biotic reactors (Fig. 3a). Similarly, there was a low *PR* (<0.13 mm y^–1^) in both the abiotic and biotic reactors (Fig. 3b)^25^. Further analysis of the surface profilometries in Fig. 2, allowed a quantitative determination of the *PR* Fig. 3b of the CS coupons. For this study, it was not possible to quantitatively determine *PD* values, due to low *PR* across the coupon surfaces upon retrieval after 28 days. Some extreme values were observed in the corrosion data (Fig. 3), particularly in measurements of pit depth and surface roughness. In the biotic condition, these values reflect the inherently heterogeneous nature of MIC, where localized microbial activity, such as the formation of electroactive or sulphide-producing biofilms, can result in highly site-specific and aggressive pitting. These values are not statistical outliers but are representative of the spatial variability typically reported for MIC. In the abiotic condition, although corrosion was generally more uniform, occasional deeper surface features were also recorded. These are likely attributable to pre-existing surface irregularities or localized micro galvanic effects on the unpolished, as-received carbon steel coupons. To preserve the integrity of the dataset and reflect the surface variability observed in industry, all values, including extremes, were retained in the statistical analysis.

### Corrosion product analysis

**Fig. 4:**
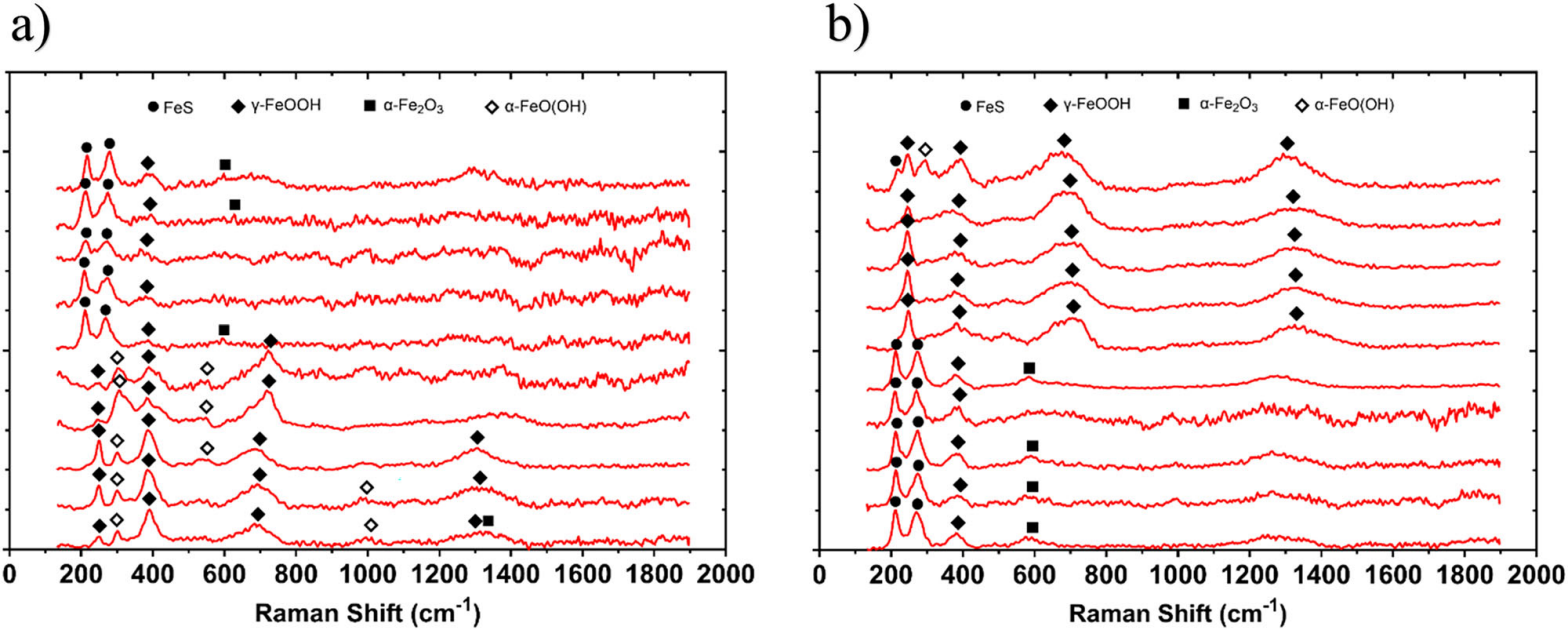
Raman Spectra of Carbon Steel After 28 days. Raman spectra of the UNS G10180 carbon steel surfaces after exposure to anaerobic nutrient-enriched artificial seawater media dosed bi-weekly with glutaraldehyde (214–250 ppm), taken on day 28. For the AR **a** abiotic and **b** biotic condition.

Analysis of the corrosion products using Raman spectroscopy are shown in Fig. 4. According to Raman bands of reference corrosion products in previous papers^26–28^, the corrosion products are identified to be primarily mackinawite (bands 208, 282 cm^–1^)^27^ for both the abiotic and biotic condition. There were also additional bands which may be attributed to sulphur, as well as reference iron oxide compounds such as magnetite, goethite, lepidocrocite, or haematite^26^. The composition of this black compact layer was identified at mid-strong bands 250, 380, 1307 cm^–1^ associated with lepidocrocite^26^. Additionally, bands at 298, 399, 481, 554, 675 and 1002 cm^–1^ have previously been shown to be associated with goethite, whilst bands at 222, 244, 298, 501, 615 and 1318 cm^–1^ are associated with haematite. Magnetite has previously been shown to be associated with bands at 675 and 550 cm^–1^ ^26–28^. The coverage of the metal sample with a black precipitate was indicative of the successful growth of corrosion products film containing ${\rm{FeS}}$ compounds. The Raman spectrum of the sample is in good agreement with literature spectra attributed to mackinawite^27^. After aging the products in air, a change in their colour from black to rust-red became visible. This is a clear indication for the oxidation of the initial products. While Raman spectra from both abiotic and biotic coupons exhibited dominant features consistent with iron oxide and oxyhydroxide corrosion products, spectra from the biotic condition also displayed additional bands in the 400–500 cm⁻¹ and approx. 1000 cm⁻¹ regions, corresponding to biofilm-associated organics or iron-sulphur species, suggesting subtle but reproducible differences in corrosion product composition under microbial influence.

**Fig. 5:**
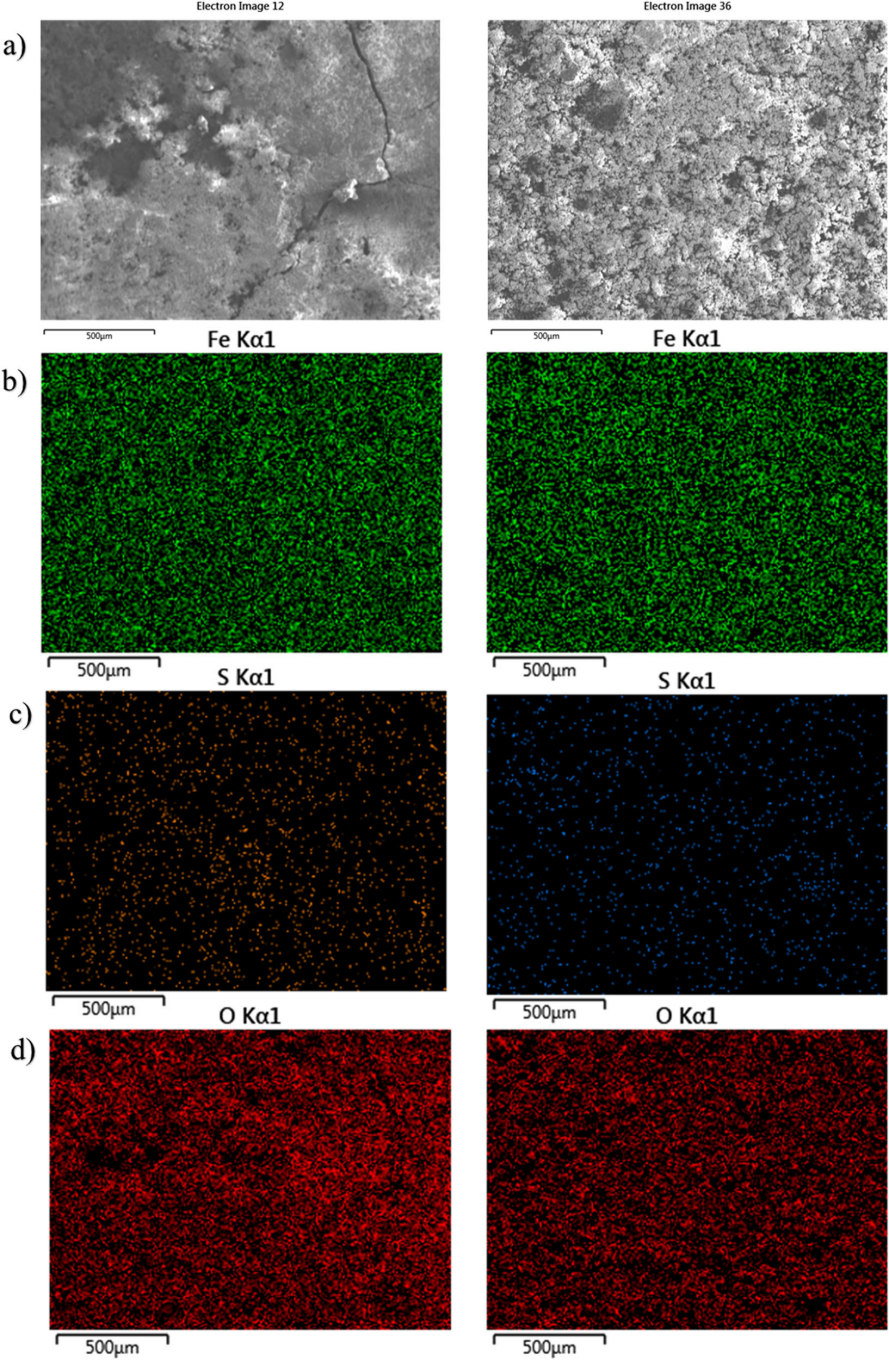
SEM-EDS elemental mapping of the UNS G10180 carbon steel, AR surfaces, after exposure to anaerobic nutrient-enriched artificial seawater media dosed bi-weekly with glutaraldehyde (214–250 ppm), taken on day 28. **a** SEM image; **b** iron map; **c** sulphur map; **d** oxygen map.

Figure 5 shows SEM-EDS elemental mapping of the UNS G10180 CS surfaces for both the abiotic and biotic conditions. Quantitative SEM-EDS data collected from elemental mapping are shown in Supplementary Table 2. The images of corrosion products and biofilms attached to the metal samples demonstrate the heterogeneity of distribution over the surface. Both conditions exhibited similar levels of surface coverage. The SEM-EDS elemental maps are shown in Fig. 5b–d. The major elements detected in coupons exposed to all conditions were ${\rm{Fe}}$, ${\rm{S}}$, and ${\rm{O}}$. Corroded areas of all coupons were mainly covered by ${\rm{Fe}}$ and ${\rm{O}}$, with heterogeneous distribution of ${\rm{S}}$. A cross-sectional image of the corrosion products was not performed.

### Electrochemical measurements

**Fig. 6:**
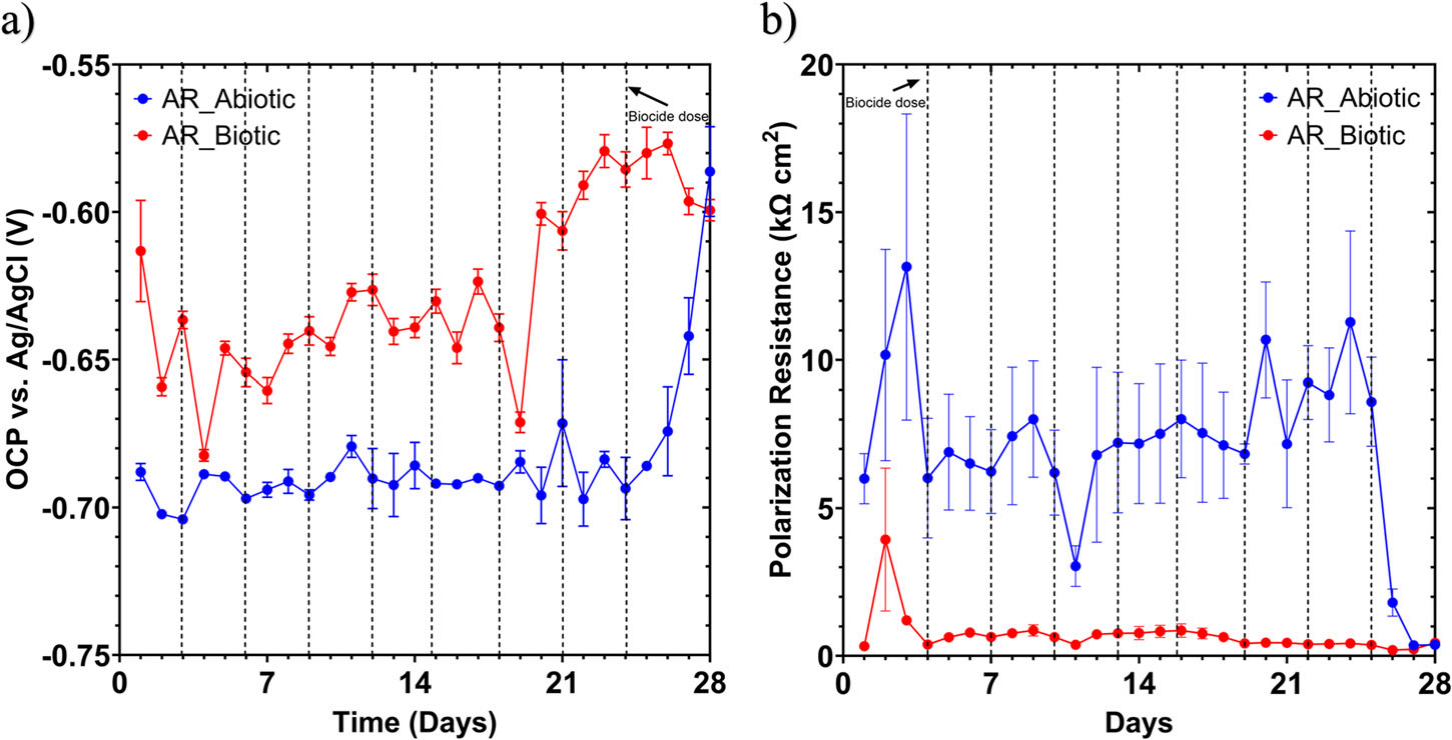
LPR data for UNS G10180 carbon steel. **a** open-circuit potentials and **b** polarization resistance in anaerobic nutrient-enriched artificial seawater media, dosed bi-weekly with 214–250 ppm glutaraldehyde (abiotic and biotic conditions), for AR coupons (data points represent mean ± standard deviation, *n* = 6). Reactor stirrer at 50 rpm.

Figure 6 shows the changes in corrosion potential (*E*_corr_) and polarization resistance (*R*_p_) between the abiotic and biotic anaerobic nutrient-enriched ASW water media, for the UNS G10180 CS coupons, and reflects the tendency of electrochemical/corrosion reactions at the metal–electrolyte interface. *E*_corr_ is the potential at which there is no net current flow, i.e., the rate of anodic metal dissolution equals the rate of cathodic reduction kinetics. Whereas, *R*_p_ reflects the charge-transfer processes at the metal-electrolyte interface (higher *R*_p_ values indicate slower corrosion kinetics and vice versa).

*E*_corr_ shifts after glutaraldehyde additions are driven by chemical and electrochemical interactions at the metallic surface for the abiotic condition and can be attributed to changes in surface chemistry, whereas there as additional influences from the biofilm structure, and microbial metabolism for the biotic condition. For the abiotic condition, Fig. 6a, there was a relatively stable *E*_corr_ until day 25, where there was a marked increase of +0.100 V. The stable *E*_corr_ is associated with a conditioning film (i.e., an adsorbed organic layer) and the formation of inorganic corrosion product layer, with no significant effect of the regular biocide additions (glutaraldehyde). A potential increase after glutaraldehyde addition can result from surface passivation, reduction in redox-active species, and complexation of metal ions. Glutaraldehyde is a reactive dialdehyde that can interact with metal ferrous cations (${{\rm{Fe}}}^{2+}$, ${{\rm{Fe}}}^{3+}$) or oxide layers to form organometallic complexes. Thus, altering the filmed CS surface, hindering dissolution and shifting the *E*_corr_ electropositively^29^. Additionally, if glutaraldehyde reduces oxidizing agents (e.g., dissolved oxygen or metal ions) or scavenges radicals in the electrolyte, the redox environment becomes less aggressive, which can also result in a more noble potentials^21,29^. Conversely, it has been reported that a decrease in *E*_corr_ after glutaraldehyde addition can also occur from pH shifts, destabilization of surface oxide layers, and electrolyte modification. Glutaraldehyde can undergo slow hydrolysis and oxidation reactions, generating acidic intermediates under some conditions. This lowers local pH, increases anodic reaction rates, and shifts the potential more electronegatively^21^. If glutaraldehyde interacts with or disrupts iron corrosion products, it can expose bare steel, accelerating anodic dissolution and decreasing *E*_corr_^21,29^. Moreover, the addition of organic compounds may alter the conductivity, dielectric constant, or ionic composition of the electrolyte, affecting the cathodic kinetics and thus influencing *E*_corr_^21,29^.

For the biotic condition, there was a gradual increase in the *E*_corr_ over the 28 days. Throughout the study, there were electronegative shifts after each biocide dosing. The changes in *E*_corr_ depend on the interplay between biocidal disruption (which suppresses electrochemical activity) and secondary effects (e.g., lysis, chemical changes in the biofilm/electrolyte interface). The potential shift is thus highly context-dependent, influenced by microbial composition, biocide concentration, exposure duration, and environmental conditions. An electropositive shift in *E*_corr_ after glutaraldehyde addition can result from a reduction in cathodic activity, biofilm disruption and reduced electron transfer, and formation of surface passivation layers. Glutaraldehyde inhibits microbial respiration, particularly in SRB and iron-reducing bacteria (IRB), which typically catalyse cathodic reactions. Inhibiting these reactions reduces cathodic depolarization, causing the potential to shift electropositively^12,30^. Additionally, biocide-induced damage to electroactive biofilms can impair extracellular electron transfer (EET) pathways, especially direct electron uptake from the metal surface. This can reduce anodic current demand and stabilize the metal surface, leading to a more noble potential^12,30^. Conversely, an electronegative shift may occur due to the release of metabolic byproducts or biofilm lysis, a localized breakdown of biofilm barriers, and a heterogeneous biofilm response to the treatment. Cell lysis following glutaraldehyde treatment may release intracellular metabolites that locally acidify the environment or complex with metal ions, accelerating anodic dissolution. Additionally, disruption of biofilm structure may expose fresh steel surface to the aggressive bulk electrolyte, increasing the anodic reaction rate and lowering the *E*_corr_. Mixed-species biofilms may contain resistant subpopulations (e.g., persister cells) or enzymatic activity that continues to drive corrosion-related redox processes after treatment, sometimes enhancing localized activity^12,30^. The potentials for both abiotic and biotic in the latter stages (Day 28) were generally similar and ranged between –0.590 V and –0.610 V *vs*. Ag/AgCl. In Fig. 6b the LPR-derived *R*_p_ remained low at approx. 600 Ω cm^2^ for the sterile abiotic condition, indicative of a uniform corrosion across a porous corrosion film. There was a marked drop after day 25 to approx. 100 Ω cm^2^. Similarly, for the biotic condition, the *R*_p_ remained low at approx. 100 Ω cm^2^. The pioneering bacterial attachment/colonisation was difficult to detect for this study. However, biofilm formation and growth kinetics will inevitably lead to a more complex electrochemical/corrosion response.

**Fig. 7:**
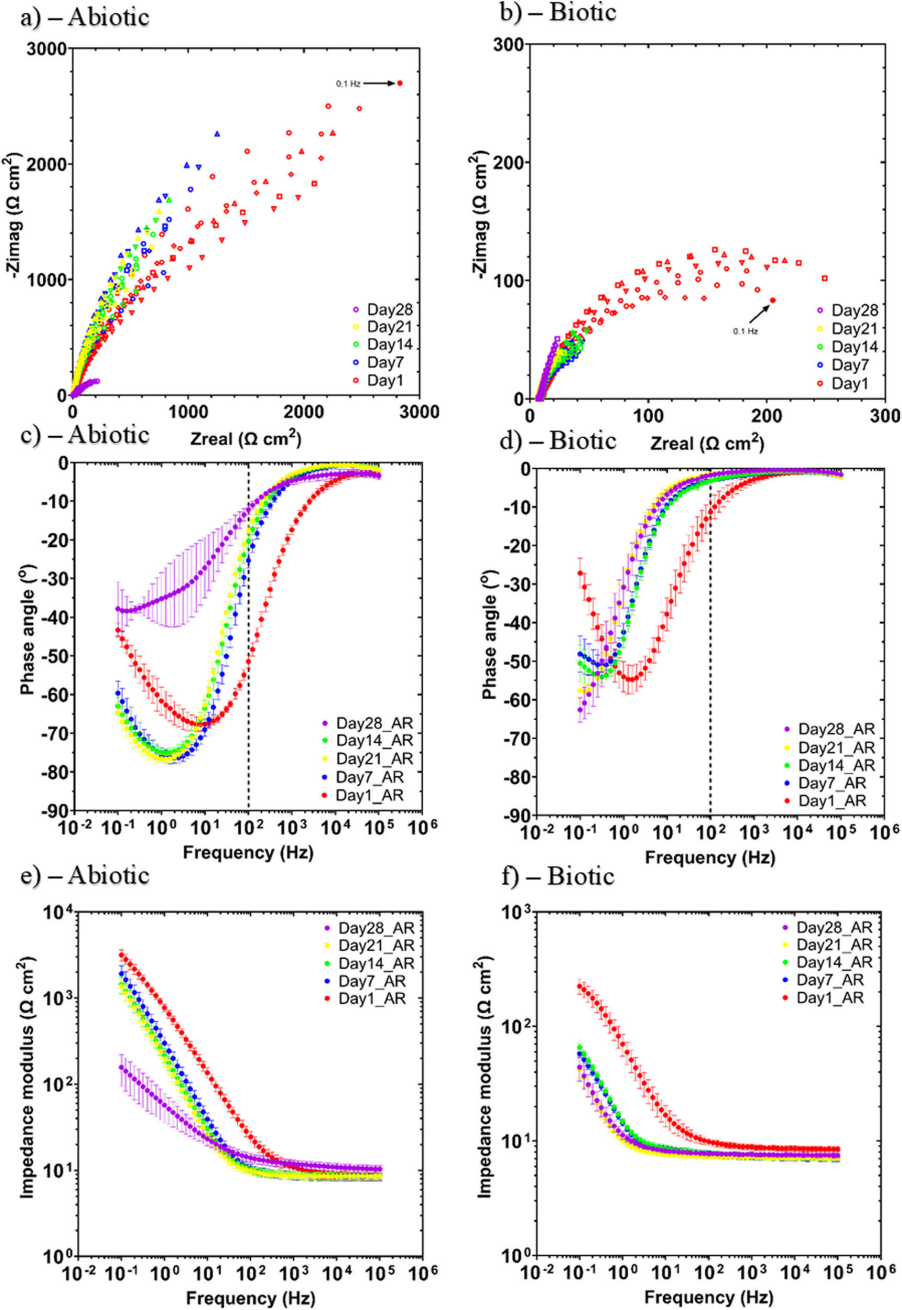
EIS data for UNS G10180 carbon steel in anaerobic nutrient-enriched artificial seawater media dosed bi-weekly with glutaraldehyde at OCP. **a**, **b** Nyquist, c, **d** Bode phase angle (${\theta }$ vs. **f**), and **e**, **f** Bode impedance modulus ( | Z| vs. f) over 28-days. (*n* = 6). Reactor stirrer at 50 rpm.

Figure 7 shows the EIS data for UNS G10180 CS in the anaerobic nutrient-enriched ASW media graphically presented in three forms: Nyquist, Bode phase angle and Bode impedance modulus plots. The sterile abiotic condition on Day 1 typifies an electrochemical response for the formation of a porous interface, with diffusion of soluble electroactive species across an organic conditioning film^31^ and a nascent inorganic corrosion product layer. The diffusive behaviour is associated with linear features having a roughly 45° slope (a Warburg impedance response) and phase angles close to 45° in low frequency region (10^–2^–10^0 ^Hz), see Figs. 7a and 7c. At Day 28, a depressed Nyquist semicircle and phase angles tending towards zero are evident, indicative of a more prominent resistive component operating within the low-frequency region. Subsequently, the abiotic impedance spectra shift towards lower frequencies (10^–2^–10^0 ^Hz), with a combined diffusive/resistive behaviour. Equally, the biotic condition had a consistently uniform EIS response over the 28-day test, with only minor variation in the spectra attributed to the biocide.

Notably, after Day 1 there are no discernible Nyquist semicircles (Fig. 7b). Here a wider low frequency region (10^–1^–10^2^) is likely to be subject to a greater influence of adsorption processes, associated with the adhesion of the pioneering bacteria on a conditioning film^32–34^ and biofilm formation. From the impedance modulus plots (Fig. 7e and f), we can see a slight difference over time at the lower frequencies. At low frequencies, the impedance reflects processes with slower dynamics, such as charge transfer/polarization resistance and mass transfer. The biotic condition appears to have increased charge transfer and polarization after the first week. Whilst the abiotic condition does not exhibit a discernible increase until day 28. The EIS spectra were fitted using an equivalent circuit model (ECM) shown in Supplementary Fig. 3. *R*_s_, *R*_film_ and *R*_ct_ are the solution resistance, the resistance of the biofilm or the corrosion product film, and the charge transfer resistance, respectively. The constant phase element (CPE) characterises the ‘non-ideal’ capacitance behaviour of either the biofilm or the corrosion product film layer, and the charge transfer capacitance. In the Supplementary Table 3, *Q* and *n*, are admittance and exponent parameters from the CPE. Both the abiotic and biotic data generally had a good fit, with the quantitative fitting results. For the abiotic control, there is a capacitive behaviour for the first three weeks, with a more prominent diffusive behaviour over the final week in the film layer (reflecting mass transfer of species across the filmed metallic interface). Whilst there was a diffusive behaviour in the double layer during the first and final week, which reflects charge transfer, due to the formation of corrosion products (rust, porous oxide layer). The exponent parameter in the double layer CPE indicates a deviation from ideal capacitive behaviour, reflecting increased heterogeneity and resistive contributions at the metal–electrolyte interface due to corrosion product buildup. There were no significant changes in *R*_ct_. *R*_film_ had a high error value, so not much could be determined from this.

For the biotic condition, there was a diffusive behaviour over the first three weeks, with a capacitive behaviour over the final week in the film layer. Conversely, there was a capacitive behaviour over the first three weeks, with a diffusive behaviour over the final week in the double layer. There are no significant changes in the *R*_ct_ in the double layer over time. The exponent parameter for the film layer is greater than 0.8 during the final week, which indicates a non-ideal capacitance response. This is true for the first three weeks in the double layer. However, during the final week of the experiment, the exponent parameter is closer to 0 for the AR coupons in the double layer, which indicates a strong diffusive behaviour. The ECM and EIS both have general agreement with the LPR data.

Supplementary Fig. 4 shows the potentiodynamic polarization curves for UNS G10180 CS for the abiotic and biotic reactors in anaerobic nutrient-enriched ASW media after 28 days (test endpoint). Supplementary Table 4 shows the corrosion parameters obtained from the polarization curves. From the Tafel slopes, limiting current behaviour is observed in the cathodic region of the abiotic condition, likely due to diffusion limitations imposed by the corrosion product layer. This can be attributed to the conditioning film and inorganic corrosion layer hindering the predominant hydrogen evolution reaction (HER) under anoxic conditions. In contrast, the biotic condition does not exhibit a clearly defined limiting current, suggesting a more mixed charge-transfer and diffusion-controlled cathodic process. This implies that the presence of the biofilm has altered the cathodic kinetics, potentially by modifying the interfacial environment and influencing the dominant reduction pathways, such as through biofilm-associated metabolic activity or surface heterogeneity. The anodic Tafel slopes (oxidation) are similar for both conditions. Overall, the abiotic condition had a higher *j*_corr_ compared to the biotic condition. This is consistent with a more uniform corrosion morphology. Similarly, the sterile abiotic condition had a more electropositive *E*_corr_ when compared to the biotic condition. It is important to note that the potential observed prior to the potentiodynamic polarizations reflects the instantaneous system response, which may differ slightly from the *E*_corr_ determined via LPR due to differences in measurement protocols and transient surface conditions at the time of acquisition. The polarization results corroborate the LPR and EIS data.

### Biofilm characterisation

**Fig. 8:**
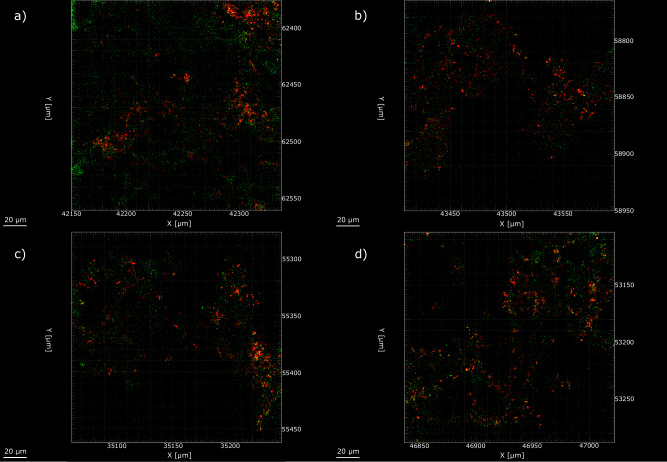
Confocal microscopy of biofilm formed over UNS G10180 carbon steel surfaces for AR. **a**, **b** biotic coupon sample 1; **c**, **d** biotic coupon sample 2, after exposure anaerobic nutrient-enriched artificial seawater media dosed bi-weekly with glutaraldehyde for 28 days.

CLSM with differentiation of live and dead biofilm cells was performed and the images can be seen in Fig. 8. The heterogeneous biofilm distribution over the surface of the CS coupons did not allow measurements of the maximum biofilm thickness. Therefore, the thickness of biofilms was not determined. From the images captured, there was a live/dead cell ratio of approximately 41% live to 59% dead.

**Fig. 9:**
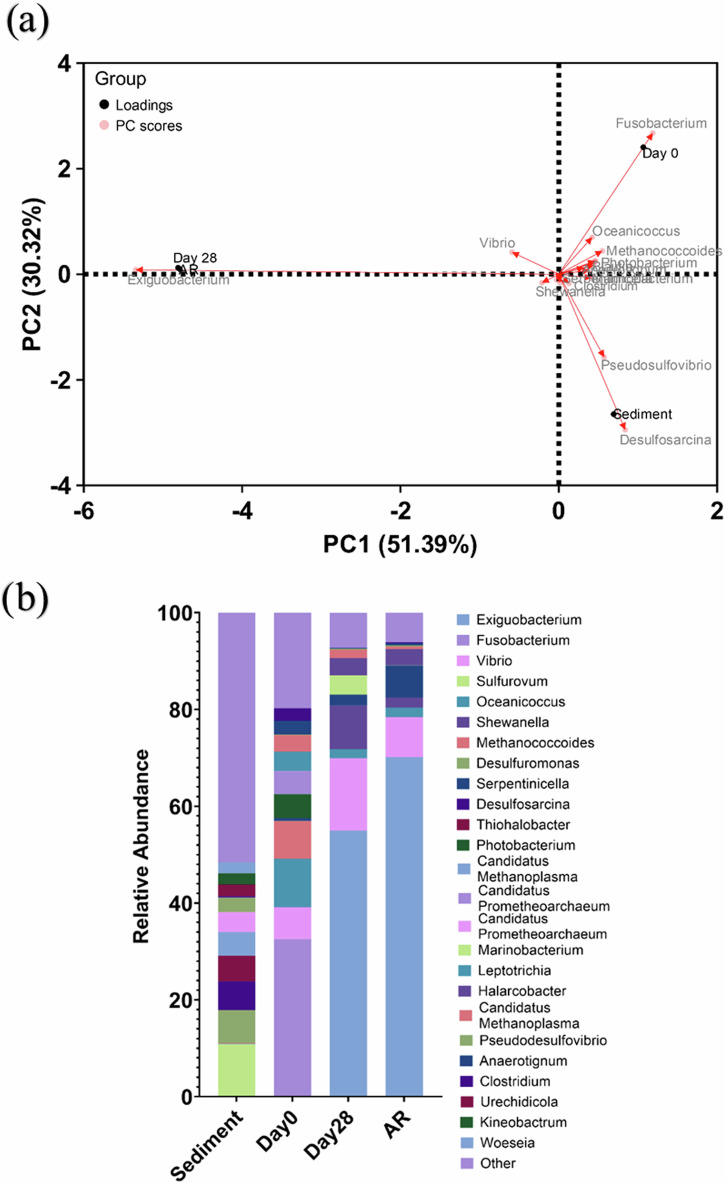
Principal Component Analysis biplot. **a** Microbial community. The results show the mean relative abundances of microbial communities classified at the genus level, for the top 25 genera, from 16S rRNA amplicon sequencing (**b**); for environmental marine sediment, Day 0, and Day 28 planktonic samples, and AR biofilms, after exposure anaerobic nutrient-enriched artificial seawater media dosed bi-weekly with glutaraldehyde for 28 days.

Active microorganism evaluation of the environmental marine sediment, the initial and final biotic nutrient-enriched ASW media planktonic samples (Day 0 and Day 28), and the biotic AR biofilm was undertaken via 16S rRNA amplicon sequencing with two target regions, V3-4 for bacteria and archaea. A total of 3,586,285 high-quality sequences were obtained after bioinformatics processing of the raw reads. From these, 97% were classified for the sediment sample, with 99.99% classified for the Day 0 planktonic sample and 100% classified for the Day 28 planktonic sample and AR biofilm sample. These sequences were taxonomically classified into microbial genera. The top 25 microbial genera are presented in Supplementary Table 5 in the supplemental material. Figure 9 summarises the sequencing data, showing a PCA (a) and a stacked bar plot (b) illustrating the relative abundances for the top 25 genera. Most genera had low relative abundances less than 2%. The dominant genera included *Sulfurovum*, *Candidatus Prometheoarchaeum*, *Candidatus Methanoplama*, *Desulfosarcina*, *Desulfuromonas* and *Thiohalobacter*. Interestingly, there were relatively high numbers of archaea in the sediment sample compared to the other samples. The sediment sample had a negative Spearman correlation coefficient (Supplementary Fig. 5) with the other samples which was attributed to changes in conditions such as temperature and media composition from the natural marine environment.

There was much less diversity in the Day 0 sample, with *Sulfurovum*, *Candidatus Prometheoarchaeum*, *Candidatus Methanoplama*, and *Thiohalobacter* all exhibiting negligible relative abundances. Whilst genera from *Fusobacterium*, *Vibrio*, *Oceanicoccus*, and *Methanococcoides* made-up approximately 55% of the relative abundance. Genera from *Photobacterium*, *Blautia*, *Leptotrichia*, *Maridesulfovibrio*, *Anaerotignum* and *Clostridium* made-up another approximately 20% of the relative abundance. Again, the Day 0 planktonic sample had mostly negative Spearman correlation coefficients with the other samples. After 28 Days, there was a significant shift in the microbial composition, with substantially lower abundances of methanogenic species. *Exiguobacterium*, *Vibrio*, and *Shewanella* were the dominant genera making up approximately 80% of the relative abundance. The Day 28 planktonic sample had a Spearman correlation coefficient of -0.47 with the sediment sample, 0.15 with the Day 0 planktonic sample and 0.88 with the AR biofilm sample. *Sulfurovum*, *Candidatus Prometheoarchaeum*, *Candidatus Methanoplama*, and *Thiohalobacter*, which were the dominant genera from the sediment sample, all had negligible relative abundances in the AR biofilm sample and Day 28 planktonic sample. The dominant genera included *Exiguobacterium*, *Vibrio*, and *Serpentinicella*, making up approximately 85% of the relative abundance. *Shewanella* also maintained a relative abundance of 2%. There were no methanogenic archaea in the biofilm sample.

The microbial activity was determined by the ATP concentrations (dissolved, dATP) in the bulk fluid, see Supplementary Fig. 6. The ATP assay did not measure any ATP from the biofilm sample. The biofilm may have been removed through the activity of glutaraldehyde. For the biotic nutrient-enriched ASW media (bulk fluid), a statistically significant increase in dATP concentration was observed when comparing Day 0 and Day 28, with average values increasing from 24.9 pg mL⁻¹ on Day 0 to 86.4 pg mL⁻¹ on Day 28 (P < 0.05). indicating active biofilm development over the course of the experiment. This indicates active microbial metabolism and suggesting the potential for continued biofilm development beyond the experimental timeframe. ATP assays conducted prior to sequencing confirmed that samples from the abiotic reactor were below the detection threshold of the negative control, indicating no biological activity and therefore no DNA was recovered for downstream analysis.

## Discussion

**Fig. 10:**
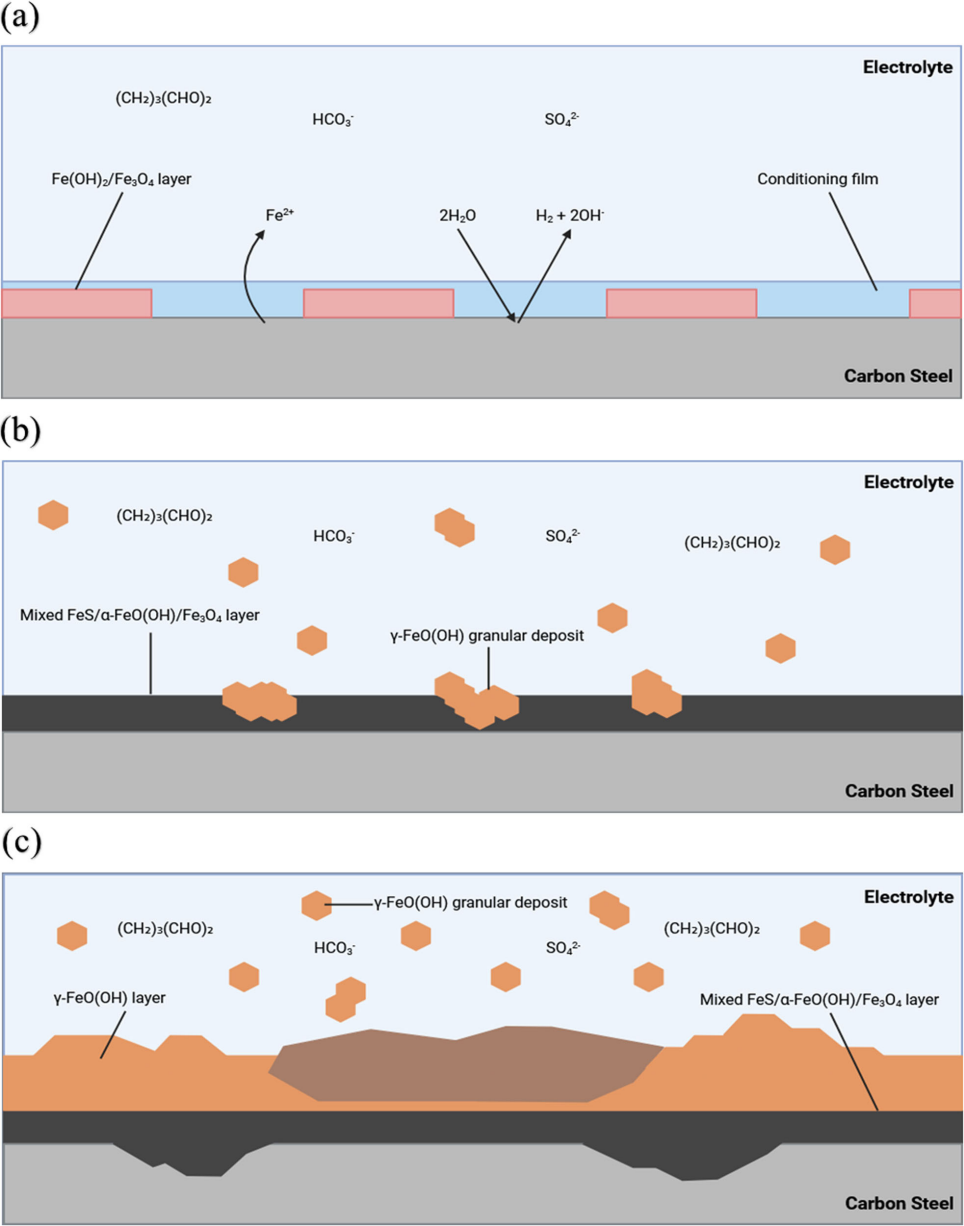
Illustration of the initial stages for UNS G10180 carbon steel in anaerobic abiotic and biotic artificial seawater media. Corrosion mechanisms, **a** the formation of nascent inorganic corrosion film and the organic conditioning film; **b** maturing corrosion film under the abiotic condition; **c** moderate uniform and pitting corrosion under patchy corrosion deposits, with increasing granular deposits. BioRender.com (2024).

This current study is focused on understanding the efficacy of a biocide, glutaraldehyde, at mitigating MIC caused by a mixed-species biofilm cultured from a littoral marine sediment. The frequency and dosage concentration were determined by reported best practices within the offshore oilfield industry. The persistence of biofilm-associated corrosion observed in this study, despite glutaraldehyde dosing, likely reflects the protective role of biofilm structure and the survival of metabolically quiescent or functionally redundant microbial populations, which are not readily inactivated by conventional biocide strategies optimized for planktonic cells. Figure 10 provides an illustration of the proposed corrosion mechanisms for the abiotic condition during the initial stages, as they evolved over time during the present study. Overall, the primary corrosion mechanisms under the abiotic condition were attributed to the formation of iron oxide corrosion products, associated with the reddish-brown film layer observed on the coupon surfaces. These were identified as magnetite, goethite, lepidocrocite, or haematite. Lepidocrocite has been identified to form in the outer layer on CS coupons permanently immersed in natural seawater^26,35^. It is important to note that oxygen, a key driver of abiotic corrosion, was intentionally excluded from this system to simulate strictly anaerobic environments relevant to many MIC scenarios. While this design enables focused investigation of anaerobic corrosion mechanisms, it limits direct comparison to oxygen-rich conditions commonly encountered in early-stage corrosion or surface-exposed infrastructure. The delayed formation of corrosion products under these conditions reflects the slower kinetics of electron acceptor-limited corrosion.

Generally, the sterile abiotic condition had a pseudo-steady state up until day 25. Then, there was a swift decrease in *R*_p_ after day 25. Overall, this may be attributed to the presence of a conditioning film and the formation of an inorganic corrosion product layer. Upon dismantling of the reactors, the abiotic surfaces were covered in a reddish-brown corrosion product. Similarly, the abiotic reactor ASW media was reddish-brown in colour. The primary corrosion products for the abiotic condition were attributed to iron oxide compounds such as magnetite, goethite, lepidocrocite, or haematite. Under abiotic conditions, mineral formation is driven by purely chemical reactions between the artificial seawater and the CS coupons. Mackinawite typically forms under highly reducing conditions, and its presence suggests a chemical reaction between dissolved sulphide and iron ions^27,28^. Mackinawite is highly reactive and accelerates corrosion by creating localized galvanic cells^27^. Lepidocrocite forms as an initial corrosion product in environments with high chloride concentrations, such as in ASW^26^. Its presence in an anoxic condition is somewhat unusual, as it typically forms in oxidizing environments. It may indicate fluctuations in localized oxygen availability or a transitional phase. Overtime, goethite is a more stable iron oxyhydroxide^36^. Magnetite is a common product in anaerobic corrosion. It acts as a protective layer, slowing down uniform corrosion, but may also promote pitting if disrupted^35,37^. From the ECMs, there is initially a capacitive behaviour over the first three weeks, which reflects ion adsorption and the development of a nascent iron oxide film. Subsequently, there was a diffusive behaviour during the final week in the film layer. Moreover, there was a diffusive behaviour in the double layer during the first and final week, which reflects charge transfer, due to the formation of corrosion products such as lepidocrocite^26^. Generally, there were low levels of uniform (*CR* between 0.025 and 0.12 mm y^–1^) or localised pitting corrosion (*PR* < 0.13 mm y^–1^) present for the abiotic condition. Previously, when investigating corrosion in the same conditions, but without the addition of a biocide, we observed a severe *CR* (>0.25 mm y^–1^) and a severe *PR* (>0.38 mm y^–1^) in the abiotic condition^22^. Interestingly, whilst both *CR* were similar, there was a significantly lower uniform *CR* for the abiotic condition when compared to the biotic condition. This can be attributed to the presence of a biofilm for the biotic condition.

For the biotic reactor, a black surface film was present on the steel coupons on day 1, immediately after inoculation with the pre-culture. Then, during the initial three-day batch phase, there was an electronegative shift observed in the *E*_corr_, which was attributed to biofilm formation on the CS coupons. After the flow of fresh media on day 4, there was a gradual increase in the *E*_corr_ associated with anodic polarization, suggesting increasing electrochemical activity, likely due to biofilm maturation and progressive microbial colonisation at the metal surface. Interestingly, up to day 21, electronegative shifts were observed following each biocide dose, coinciding with reductions in ${{\rm{H}}}_{2}{\rm{S}}$. This trend implies that the biocide disrupted microbial sulphate reduction and associated EET, temporarily suppressing anodic dissolution and shifting the corrosion potential in the negative direction. However, after day 21, each subsequent biocide application resulted in electropositive shifts a pattern also observed in the abiotic control. This shift may reflect a change in the dominant surface processes and chemical interactions at the metal interface. One likely explanation is that cumulative biocide exposure altered the physicochemical environment, either through surface passivation, modification of corrosion product layers, or accumulation of reactive aldehyde-metal complexes, leading to a temporary reduction in active anodic dissolution. In the abiotic system, the absence of microbial metabolism suggests this electropositive trend arises from purely chemical phenomena, such as the formation of glutaraldehyde-iron complexes. These changes can reduce the electrochemical driving force for corrosion, resulting in a more noble *E*_corr_. Together, these findings suggest a time-dependent shift in the dominant mechanisms of corrosion, from biologically mediated processes to chemically influenced corrosion dynamics, as the system progresses and the cumulative effects of repeated biocide exposure manifest. The biotic condition generally had a consistently uniform EIS response over the 28-day test, with only minor variation in the spectra attributed to the biocide. Additionally, the *R*_p_ was generally low for the biotic condition. For the biotic condition, there is initially a diffusive behaviour over the first three weeks. This is indicative of a non-ideal capacitance response. Whilst there is a capacitive behaviour over the final week in the film layer, which reflects ion adsorption and the development of a film layer. Conversely, there is a capacitive behaviour over the first three weeks, with a diffusive behaviour over the final week in the double layer. This indicates a strong diffusive behaviour and reflects charge transfer due to the formation of corrosion products. The coverage of the metal sample with a black precipitate was indicative of the successful growth of a corrosion products film containing ${\rm{FeS}}$ compounds. However, after aging the products in air, a change in their colour from black to rust-red became visible. This is a clear indication for the oxidation of the initial products. The composition of this corrosion layer was associated with lepidocrocite^26^, as well as mackinawite^27^. Under biotic conditions, the presence of biofilm fundamentally alters the corrosion process due to the microbial activity of heterotrophic microbes. SRB can play a critical role in mackinawite formation by reducing sulphate to sulphide, which reacts with iron. The biofilm can concentrate sulphides, accelerate mackinawite formation, and lead to more rapid substrate degradation. Moreover, the presence of SRB could suggest enhanced localised pitting corrosion over time^38–40^. Microbial activity can also facilitate the oxidation of ferrous ions, even in anaerobic environments, through microbially mediated redox gradients within the biofilm. Lepidocrocite and goethite may form as transient phases or in microaerophilic niches within the biofilm where oxygen diffuses slowly from the surrounding water. Their presence could indicate microbial consortia involving iron-oxidizing or nitrate-reducing bacteria^36^. Magnetite formation in the biotic condition could result from microbial reduction of ferric oxides. Biofilms can facilitate this by creating microenvironments with varying redox potentials and creating highly localized environments with steep pH and redox gradients, promoting the coexistence of minerals that typically require different conditions. Microbial activity can exacerbate corrosion processes, leading to more aggressive and localized pitting corrosion over time^41^. While similar mineral phases may form in both abiotic and biotic conditions, the presence of biofilms can exacerbate corrosion over time, allowing for more diverse corrosion mechanisms, and creating a more complex and aggressive environment.

Microscopic analysis provided further insights into the surfaces of the CS coupons. The heterogeneous biofilm distribution over the surface of the CS coupons was captured using CLSM. From the images, it was clear to see that the biocide had a clear impact on the survivability of the biofilm. There was a live/dead cell ratio of approximately 41% live to 59% dead. Once the surfaces had been cleaned, surface profilometry analysis revealed that there were low levels of uniform (*CR* between 0.025 and 0.12 mm y^–1^) or localised pitting corrosion (*PR* < 0.13 mm y^–1^) present for both the abiotic and biotic conditions. It should be noted that only 2–3% of the analysed surfaces observed pitting. However, after 28 days, the biotic condition did exhibit pits with a greater average area. There was approximately a 3.7× increase in pit area. This observation, of larger average pit areas under biotic conditions, is consistent with established characteristics of MIC. Unlike abiotic corrosion, which typically results in more uniform metal degradation or smaller, evenly distributed pits, MIC often drives highly localized attack due to the formation of metabolically active biofilms. These biofilms generate microenvironments with steep chemical and electrochemical gradients, facilitating localized acidification, accumulation of corrosive metabolites such as ${{\rm{H}}}_{2}{\rm{S}}$, and disruption of protective oxide layers. Additionally, electroactive microorganisms can directly interact with the metal surface through EET, enhancing anodic dissolution at specific sites. These localized processes lead to the formation of deeper and broader pits. As such, the pit morphology observed in this study is characteristic of localised pitting caused by biofilms^42,43^. Previously, when investigating corrosion in the same conditions, but without the addition of a biocide, we observed a moderate *CR* (between 0.025 and 0.12 mm y^–1^) and a severe *PR* (>0.38 mm y^–1^) in the biotic condition^22^. Typical corrosion rates of carbon steel in anoxic environments are reported to be between 0.2 and 0.4 mm y^–1^ ^44^. While there is certainly an argument that the biocide is decreasing the overall incidence of pitting and the rate of pitting, compared to the previous experiment, there remains a threat of MIC as the microorganisms are not completely eradicated/removed. Over time, biofilm formation can exacerbate MIC. If microorganisms are allowed to form thick biofilms, this can ultimately result in higher corrosion rates due to microbial corrosion mechanisms, which generate anodic and cathodic regions that affect the passive film on the material surface^22^. Thus, the biocide dosage and frequency tested in this experiment are insufficient at mitigating microbial growth. Whilst localised pitting corrosion did not appear to be significantly exacerbated by the biotic condition, longer-term studies may reveal critical stages of biofilm maturation and corrosion progression. For this study, it was not possible to quantitatively determine *PD* values.

Analysis of the community dynamics revealed a marked change in the predominant relative abundances of microorganisms. The dominant genera from the sediment sample were generally anaerobic, halophilic, and obligately chemolithoautotrophic. They collectively play crucial roles in the biogeochemical cycles of carbon, sulphur, and methane in marine environments. There was much less diversity in the Day 0 sample, with Fusobacteriota, Proteobacteria, and Clostridia classes making up approximately 75% of the relative abundance. *Fusobacterium*, *Vibrio*, and *Oceanicoccus* alone made-up approximately 55% of the relative abundance. *Fusobacterium* is primarily known as a genus of bacteria that inhabit animal guts. They are fermentative bacteria that have been reported to produce butyrate and other short-chain fatty acids as metabolic byproducts^45^. *Vibrio* sp. are facultative anaerobes, known for their ability to form biofilms on various surfaces, including metals^46^. While *Oceanicoccus* sp. are relatively undocumented^47^. They likely contribute to the degradation of organic material in marine ecosystems. Genera from *Photobacterium*, *Blautia*, *Leptotrichia*, *Maridesulfovibrio*, *Anaerotignum* and *Clostridium* made-up another approximately 20% of the relative abundance. These genera represent a diverse group of bacteria contributing to processes such as fermentation, and sulphur cycling in marine environments^48,49^. *Exiguobacterium*, *Vibrio*, and *Shewanella* were the dominant genera, making up approximately 80% of the relative abundance in the bulk fluid. *Exiguobacterium* sp. are facultative anaerobes, capable of utilizing a variety of organic compounds for energy. They are known for their ability to survive under extreme environmental conditions, such as high salinity. In marine environments, *Exiguobacterium* contributes to the degradation of organic material^50^. Similarly, for the AR biofilm sample, the dominant genera included *Exiguobacterium* and *Vibrio*. *Serpentinicella* also saw an increase in relative abundance. *Serpentinicella* represents a group of extremophilic bacteria which have been reported to be adapted to extreme environments characterised by high pH (alkaline conditions) and low availability of organic carbon^51^. Previously, when investigating corrosion in the same conditions, but without the addition of a biocide, *Malaciobacter*, *Crassaminicells*, *Maridesulfovibrio*, and *Halarcobacter* made up approximately 50% of the relative abundance in the biofilm samples^22^. Together, these bacteria contribute to maintaining the balance of nutrients and energy flow in marine environments. *Vibrio* and *Serpentinicella* were also present in low relative abundance, but *Exiguobacterium* was not present in the top 25 genera. There was also an increase in the relative abundance of electroactive bacteria, such as *Desulfomicrobium*, *Shewanella*, and *Desulfuromonas* in the biofilm samples. These electroactive bacteria have been reported to play a key role in EET^52^, an important process in MIC. Thus, the biocide appears to have an impact on the overall the community dynamics within the biofilm, causing a shift in the predominant species present. Species adapted to extreme environments, such as *Exiguobacterium* and *Serpentinicella* and species characterised as employing biofilm phenotypes, such as *Vibrio*, appear to be enhancing chemical tolerance to glutaraldehyde. Alternatively, these species enable increased survival/recalcitrance through uncharacterised inhibition resistance mechanisms. Interestingly, for this study, *Shewanella* also maintained a relative abundance of 2% in the biofilm sample. *Shewanella* sp. are widely distributed in marine environments and play a crucial role in the biogeochemical cycling of elements like iron and manganese in marine sediments. Additionally, *Shewanella* has been reported to play a key role in EET^53^.

**Fig. 11:**
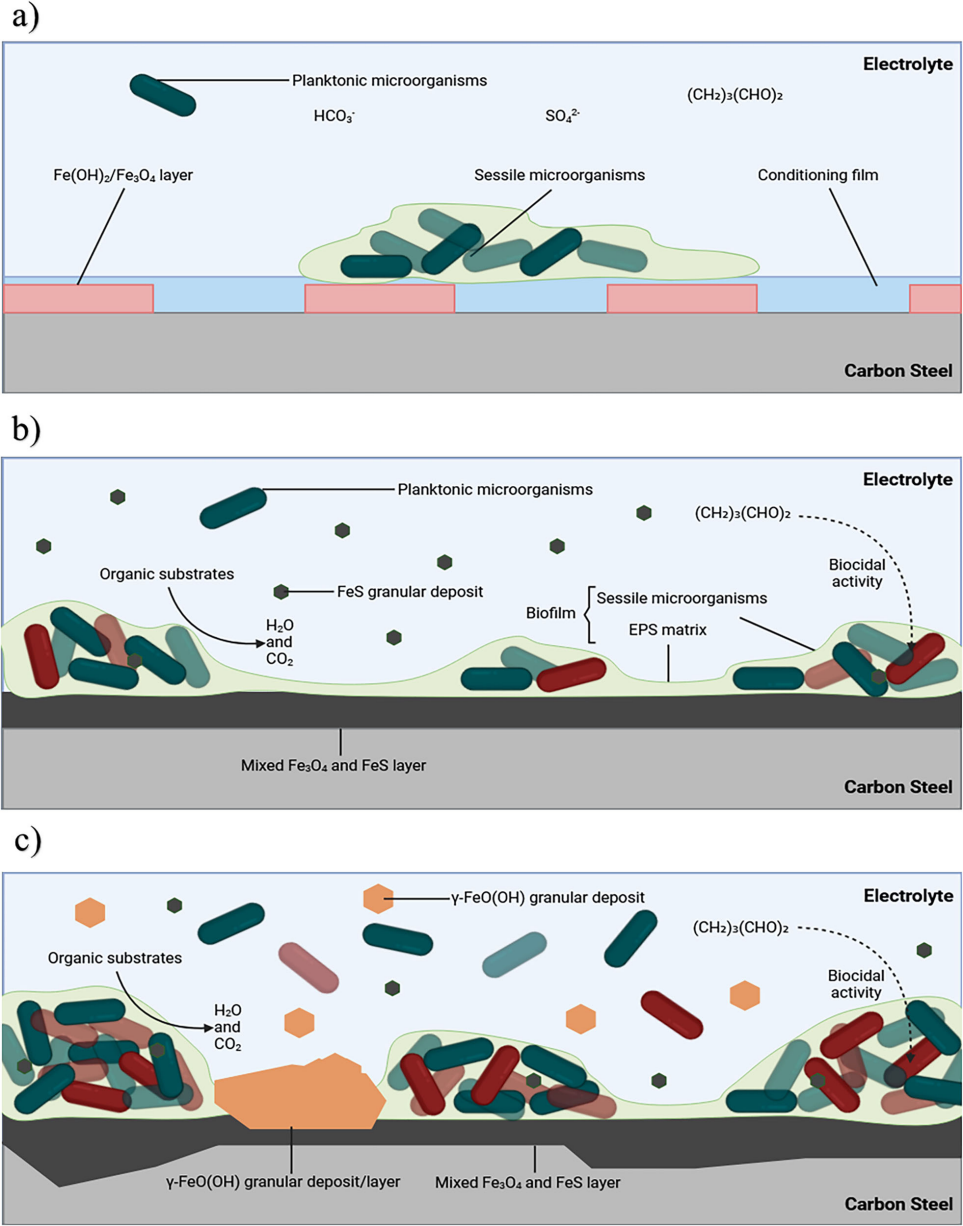
Illustration of the initial stages for UNS G10180 carbon steel in anaerobic abiotic and biotic artificial seawater media. Corrosion mechanisms, **a** the formation of nascent inorganic corrosion film and the organic conditioning film with pioneering bacterial attachment; **b** maturing corrosion film with inhibited biofilm growth and colonisation due to biocidal activity; **c** moderate uniform and pitting corrosion under patchy corrosion deposits, with increasing granular deposits, as well as patchy biofilm due to biocidal activity. BioRender.com (2024).

For this study, the electrochemical response under both abiotic and biotic conditions was distinct. For the biotic condition, whilst a reddish-brown corrosion product was also observed, this was largely a granular deposit that easily sloughed off the surface. Underneath the granular deposit was a black deposit that more closely adhered to the surface. The composition of this corrosion layer was associated primarily with mackinawite^27^. Figure 11 proposes biotic reaction mechanisms, associated with mackinawite formation, as well as iron oxide corrosion products.

As stated earlier, the mixed-species biofilm contained *Exiguobacterium* and *Serpentinicella*, which are known for their ability to survive under extreme stress, and in environmental conditions not conducive to growt,h such as high salinity. Critically, the mixed-species biofilm also contained *Vibrio* known for their ability to form biofilms on various surfaces, including metals. It is hypothesised that these genera were working synergistically to enhance chemical tolerance towards glutaraldehyde. Alternatively, the biofilm phenotype enabling increased survival/recalcitrance. Glutaraldehyde is known to kill bacteria by cross-linking proteins, inactivating enzymes, disrupting cell membranes, and damaging DNA. These combined effects lead to the disruption of essential cellular processes, resulting in bacterial death^52^. However, the formation of a biofilm can enhance chemical tolerance. The biofilm acts as a diffusion barrier, which limits the efficacy of biocidal treatments. Moreover, genetic adaptation and the presence of resistant species, such as *Exiguobacterium* and *Serpentinicella* may have been key determining factors. The cyclic biocide treatment protocol employed failed to completely eradicate the mixed-species biofilm community. The biocidal efficacy observed in this study aligns with trends reported in previous literature^1,2^, showing that glutaraldehyde, particularly at lower concentrations, can reduce but not fully eliminate biofilm-associated microorganisms. Prior studies have demonstrated that glutaraldehyde is less effective against established, mixed-species biofilms than against planktonic cells. In line with these findings, our results show a measurable reduction in ${{\rm{H}}}_{2}{\rm{S}}$ production and transient shifts in electrochemical behaviour following treatment, indicating partial disruption of microbial activity. However, complete eradication of the biofilm was not achieved, consistent with reports that sessile communities often exhibit increased resistance due to protective EPS, reduced biocide penetration, and the presence of tolerant subpopulations. Our study extends this understanding by applying glutaraldehyde in a dynamic, mixed-species system under flow, simulating more realistic operational conditions. These results reinforce the need for complementary treatment strategies and support the growing consensus that evaluating biocides against mature biofilms in complex systems is essential for generating industry-relevant performance data. It is hypothesised that longer-term studies may reveal critical stages of biofilm maturation and corrosion progression.

Comparison of the current study with previously published experiments^22^ using the same dual-reactor model without glutaraldehyde treatment provides additional insight into how biocide exposure influences MIC progression. In the absence of glutaraldehyde, biotic reactors exhibited higher pit density and greater microbial activity, particularly associated with electroactive SRB and IRB, while abiotic controls showed more uniform corrosion and shallower pitting. In the present study, the addition of glutaraldehyde to the biotic system resulted in transient suppression of ${{\rm{H}}}_{2}{\rm{S}}$ production and electrochemical shifts, suggesting partial inhibition of microbial metabolism and altered corrosion dynamics. Notably, pit morphology and electrochemical responses in the biocide-treated reactors showed a trend toward reduced corrosion activity compared to untreated biotic conditions, though not equivalent to sterile abiotic controls. While these findings suggest glutaraldehyde can modulate MIC under the tested conditions, the absence of full biological replication limits definitive conclusions regarding reproducibility. Nonetheless, the availability of data from four distinct conditions, biotic/abiotic with and without biocide, provides a valuable comparative framework and supports the further development of standardized protocols for MIC biocide efficacy assessment.

A limitation of this study is the absence of endpoint pH measurements. Future experiments will incorporate final pH assessments to better contextualize microbial activity and corrosion behaviour under anoxic conditions. Additionally, a key limitation of this study is the absence of biological replication, as only a single biotic and abiotic reactor was operated in parallel over 28 days. While multiple coupons provided within-system replication, the lack of independently repeated experiments limits our ability to assess inter-experimental variability and the broader reproducibility of the observed trends. This constraint reflects the substantial time and cost associated with operating complex, long-duration biofilm reactor systems; however, future studies will prioritize full biological replication to enhance statistical power and validate these findings across multiple experimental runs. Additionally, future studies should also aim to capture the full ecological complexity of estuarine biofilms by incorporating microaerophilic and aerobic community members that naturally contribute to oxygen scavenging, metabolic cross-feeding, and co-factor provisioning processes that may be disrupted in strictly anaerobic systems but are likely critical to the synergistic interactions driving biocorrosion. Incorporating aerobic controls or redox gradient transitions would provide a more comprehensive view of the corrosion continuum and enhance relevance to field conditions where oxygen availability may fluctuate.

This study demonstrates the utility of a dual anaerobic biofilm reactor system for evaluating the efficacy of glutaraldehyde treatment against mixed-species biofilms under simulated marine corrosion conditions. While glutaraldehyde application led to measurable reductions in ${{\rm{H}}}_{2}{\rm{S}}$ concentration and transient shifts in *E*_corr_, it did not fully eradicate the biofilm community, highlighting the limitations of cyclic biocide dosing when used in isolation. Notably, electrochemical and imaging data suggest that biofilm structural heterogeneity and species-specific adaptations can contribute to the persistence and resilience of the microbial community.

- The electrochemical responses differed significantly between abiotic and biotic conditions. Under biotic conditions, electronegative shifts were observed following each biocide dose, alongside a decrease in ${{\rm{H}}}_{2}{\rm{S}}$ concentration after each application.
- The primary corrosion product identified for the biotic condition was mackinawite. There were also additional bands which may be attributed to sulphur, as well as reference iron oxides, such as magnetite, goethite, lepidocrocite, and haematite, for both conditions.
- Overall, a moderately low uniform corrosion rate and minimal localized pitting were observed for both conditions. Analysis of pit densities was not feasible due to the low pit rate across the coupon surfaces. However, the biotic condition did exhibit pits characteristic of MIC.
- Finally, biofilm characterisation using sequencing demonstrated that *Exiguobacterium*, *Vibrio*, and *Serpentinicella* all appeared to play a critical role in enhancing chemical tolerance to glutaraldehyde or alternatively enabling increased survival/recalcitrance.

While this study does not claim to fully optimise biocidal treatment strategies, it successfully established a reproducible experimental framework to evaluate biocide efficacy under environmentally relevant MIC conditions, over extended exposure times of four weeks. The dual-reactor approach provides fresh insights into key biocide dynamics, including incomplete suppression of microbial activity, delayed electrochemical effects, and persistent localized corrosion. These findings underscore the need for a better understanding of biofilm behaviour under biocidal stress and support the development of MLOE protocols to assess treatment efficacy. By integrating microbiological, electrochemical, and surface characterization data, our approach provides a reproducible framework for testing and optimizing biocidal strategies that account for the complex nature of MIC. Importantly, the methodology and findings from this work are being incorporated into ongoing efforts to define standardized biocide testing protocols under the guidance of the Energy Institute and the Association for Materials Protection and Performance (AMPP; SC-22 Biodeterioration). Thus, this research contributes directly to the translation of laboratory science into practical guidelines for MIC mitigation, bridging the gap between science, standards, and industry impact.

## Methods

### Test conditions

Two anaerobic CDC (Center for Disease Control) biofilm reactors (Biosurface Technologies Corporation) were used: an abiotic control reactor and a biotic test reactor (key dimensions: 22 cm reactor height and 12 cm internal diameter; 21 cm coupon holder rod; 1.27 cm coupon diameter), along with Masterflex High-Performance Precision Pump Tubing, C-Flex (Model 06424), which is a Thermoplastic Elastomer (TPE) with low gas permeability. Sterile CS coupons were fixed in reactors and exposed to two separate conditions for 28 days. The comparison between abiotic and biotic corrosion was conducted under anoxic conditions to simulate marine environments where oxygen is limited or absent, such as sediments or biofouled interiors of pipelines and tanks. While this suppresses classical oxygen-driven abiotic corrosion, it enables the isolation and assessment of biofilm-mediated corrosion mechanisms. Trace residual oxygen may have been present during initial setup, as indicated by transient resazurin colouration, but was rapidly depleted following inoculation and media flow. Anoxic conditions were subsequently maintained by sparging the system with nitrogen gas (oxygen-free nitrogen) (BOC Nitrogen (Oxygen Free), 44-W) over an initial three-day batch phase. For this experiment, only nitrogen gas and not a mixture of nitrogen/carbon dioxide was used for strict oxygen removal, and to support the growth of a broad range of anaerobic microorganisms. Anoxic conditions, considered to be hypoxic or low ${{\rm{O}}}_{2}$, are characterised as a system with low concentrations ranging between 1% and 30% saturation. Strict obligate anaerobes will not survive if there is more than half a percent ${{\rm{O}}}_{2}$ in the environment, while moderate obligate anaerobes can still grow in a 2 to 8% ${{\rm{O}}}_{2}$ environment^54^. ASW (Artificial Seawater) (SwellUK, Aquarium Systems Sea Salt Instant Ocean) supplemented with yeast extract (ThermoFisher, Oxoid, Yeast Extract LP0021) and resazurin solution (0.1%, 0.5 mL L^-1^) (Merck) was used as the growth medium. The test media had the following composition: 18,740 mg L^–1^ ${{\rm{Cl}}}^{-}$, 10,454 mg L^–1^ ${{\rm{Na}}}^{+}$, 2,631 mg L^–1^ ${{\rm{SO}}}_{4}^{2-}$, 1,256 mg L^–1^ ${{\rm{Mg}}}^{2+}$, 400 mg L^–1^ ${{\rm{Ca}}}^{2+}$, 401 mg L^–1^ ${{\rm{K}}}^{+}$, 194 mg L^–1^ ${{\rm{HCO}}}_{3}^{-}$, 6 mg L^–1^ ${{\rm{B}}}^{3+}$, 7.5 mg L^–1^ ${{\rm{Sr}}}^{2+}$, and 1000 mg L^–1^ yeast extract (Supplementary Table 6). It was acknowledged that yeast extract contains redox mediators that may adsorb onto the electrode surfaces and chelate metal ions and the test matrix was designed to highlight any significant interference^55^. Resazurin was added as a redox indicator, as it is colourless under oxygen-free conditions but changes to a pink colour in an oxygen-containing environment. Agitation of the reactor baffles was set to 50 rpm to maintain a homogeneous solution. The reactor temperature was at ambient conditions (20 °C). Prior to inoculating the biotic reactor, a three-day pre-culture was prepared in a blue-cap flask (50 mL), consisting of 10% marine sediment with the remainder fresh ASW media. The biotic reactor was inoculated using a sterile syringe, where 10% of the working reactor volume (35 mL) was added as the inoculum. Initial adenosine triphosphate (ATP) measurements were taken from the pre-culture and long-term frozen stocks were prepared using 20% glycerol. Supplementary Fig. 7a shows a schematic of the full experimental setup, with Supplementary Fig. 7b illustrating the three-electrode cell setup within each anaerobic CDC biofilm reactor. Both reactors were operated in batch mode for the first three days to allow settlement and to facilitate biofilm formation in the biotic reactor. After this period, the reactors were switched to continuous flow of fresh media at a rate of 0.2 mL min^–1^, which replaced about 50% of the 600 mL total volume daily (288 mL day^–1^). The reactors were dosed twice weekly with a biocide on days 4, 7, 10, 13, 16, 19, 22, and 25, using 5 mL of glutaraldehyde (Merck, 340855) with a concentration range of 214–250 ppm. The range accounts for the variations in working volume of the reactors between 300–350 mL. The biocide had a residence time of up to 24 h, as the flow of fresh media slowly diluted the biocide. It was acknowledged that yeast extract may have partially neutralized or reduced the activity of glutaraldehyde through chemical interactions. However, the dosing strategy was designed to reflect lower-bound industrial concentrations with up to 24 h of residence time prior to dilution by fresh media.

### Microbial consortia

The sheltered zone littoral sediment microbial consortia were collected at a depth between 10–15 cm below the sediment surface during low tide from Langstone Harbour, United Kingdom (50°50'11.9“N 0°58'47.5“W). The coastal/estuarine marine sediment (very fine and cohesive mud and silt deposits) was selected to sample microorganisms living under low oxygen conditions. Although the in situ temperature of Langstone Harbour ranges from approximately 7–18 °C, the sediment inoculum was incubated at 37 °C to selectively enrich for fast-growing, metabolically active anaerobes, particularly sulphate-reducing and fermentative bacteria, to establish a robust, corrosion-relevant biofilm under controlled laboratory conditions. As such, the sediment samples were added to 500 mL of the ASW medium and stored at 37 °C in an anaerobic chamber to maximize the recovery of the diverse microbial populations. Mesophilic bacteria can survive and grow in temperatures between 10 °C and 50 °C. Thus, a tropic strategy to promote cell growth and viability was employed to maximise microbial recovery. The anaerobic chamber gas mixture consisted of 85% ${{\rm{N}}}_{2}$, 10% ${{\rm{CO}}}_{2}$ and 5% ${{\rm{H}}}_{2}$ (BOC Anaerobic Growth Mix, 290563-L). The composition of the culture medium is described above and presented in Supplementary Table 6. Long-term storage of sediment samples and microbial consortia was employed to create frozen stocks at –80 °C.

### Carbon steel coupon preparation

UNS G10180 (AISI 1018) carbon steel disc coupons (Biosurface Technologies – RD128 CS), with dimensions of 12.7 mm diameter × 3.8 mm thickness, were used as-received (AR) (*R*_a_ = 1.26765 ± 0.64544) without mechanical polishing to preserve the native surface heterogeneity representative of field-exposed infrastructure. Prior to use, the surfaces were pretreated by submerging in 70% ethanol and allowed to air dry, in a microbiological safety cabinet, under ambient conditions to ensure sterility. The surface profiles and weights for all coupon samples were assessed prior to starting the experiment, on Day 0, for surface profilometry and gravimetric analysis to be performed at the completion of the experiment after Day 28. Three-dimensional (3D) surface profiles were taken using a 3D optical profilometer (Alicona imaging infinite focus microscope IFM G4 3.5). A Mettler AT201 was used to take five measurements of the initial weights of all coupons.

### Experimental setup

Before autoclaving, the two anaerobic CDC biofilm reactors were cleaned with detergent and allowed to dry. The empty reactors with attached tubing were placed in autoclavable bags; all tube openings and air filters (Millex, 0.2 µm) were covered in aluminium foil, with tube openings clamped shut. The rods, without coupons, were also covered in aluminium foil and placed in an autoclavable bag. The empty assembled reactors were autoclaved for 15 mins at 121^o^C, along with prepared ASW test media. After cooling, the reactors were transferred into a sterilized microbiological safety cabinet, along with all rods, CS test coupons, as well as any sensors and electrodes. Working electrode rods were prepared in advance. For each working electrode rod, wires were soldered to each coupon separately. The coupon face with the soldered wire was then covered with a lacquer solution (Polishing Shop, Type 45 Stop Off Lacquer) and allowed to dry. To assemble the reactors, all rods with coupons were submerged in 99% ethanol for at least 10 s, then inserted into the autoclaved reactors. This was to disinfect the carbon steel coupons. Any sensors or electrodes used in place of a rod were also inserted, after being sterilized with 99% ethanol for at least 10 s. The medium bottles and all tubing were connected in a microbiological safety cabinet. Once both reactors were fully assembled, they were transferred to the working area, with access to a N_2_ gas supply. The tubing was evenly split into each reactor to equalize the pressure gradient caused by the peristaltic pump (Matson Marlow 300 series).

### Sulphide analysis

Sulphide concentrations were monitored daily in each reactor using a Unisense, SULF-50 sulphide microsensor (50 μm diameter) and amplifier (Unisense, ${{\rm{H}}}_{2}{\rm{S}}{\rm{UNIAMP}}$). The microsensor measures the partial pressure of ${{\rm{H}}}_{2}{\rm{S}}$ gas, and the total concentration is a function of pH and temperature. The microsensor limit of detection is 0.3 µM, with a range from 0–300 µM sulphide in water. Calibration utilised the ${{\rm{H}}}_{2}{\rm{S}}$ and SULF sensor calibration kit (Unisense, ${\rm{CALKIT}}-{{\rm{H}}}_{2}{\rm{S}}$). Due to the nature of the experimental setup, it was not possible to calibrate the microsensors during the experiment. However, calibrations were performed both prior to starting the experiment and once the experiment had finished to confirm that the sensors were still calibrated. The SensorTrace Suite software was used to collect the sulphide microsensor data. The sensor has a higher signal for zero right after it has been connected to the amplifier, thus each microsensor collected readings for five minutes (approximately 300 data points) on each day. This was to allow the sensor to stabilise.

### Surface profilometry and visual inspection

Corrosion products and biofilms were removed from the surface using the cleaning protocol described for the gravimetric analysis. Three-dimensional (3D) profiling of the CS surfaces was reconstructed using an Alicona imaging infinite focus microscope IFM G4 3.5. The images allowed assessment of changes in surface roughness compared to the surface profiles obtained prior to testing. Additionally, ImageJ/Fiji was used for the quantitative determination of pit depth, width, height, percentage area, and to assess pit rate (*PR*) and pit density (*PD*). This analysis was performed on thirty total locations on six (*n* = 6) AR coupons (five locations each). The method involved applying a colour threshold to depths greater than 5 µm. Then, the images were converted to a binary mask. Next, measurement parameters were selected for areas greater than 650 µm^2^. Finally, the images were analysed to display counts, area, and average size of pits. The pit parameters were adapted from ASTM G48-11^24^. For pit rate analysis, the deepest pits from each image were captured using the Alicona. Pit rates were calculated using the formula described in NACE SP0775-2023^25^.

### Gravimetric analysis

Corrosion products and biofilms were removed following the ASTM G1-03 standard with a 15% inhibited hydrochloric acid described in NACE SP0775-2023^25,56^. A stock solution was made of 37.5% HCl (Merck, Suprapur, 1.00318.0500) to which 10 g L^–1^ of 1,3-di-n-butyl-2 thiourea (DBT) (Merck, 8.20423.0250) was added. Immediately prior to use, the stock solution was diluted by slowly adding a measured volume of stock solution to an equal volume of deionised water with stirring. A Mettler AT201 was used to take five measurements of all coupons (*n* = 6). Corrosion rates (*CR*) were determined by the gravimetric technique that considers the weight loss and surface area of the metal samples described in NACE SP0775-2023^25^.

### Electrochemical analysis

Electrochemical measurements were performed using a Gamry Instruments potentiostat (Ref 600 Plus). The electrochemical behaviours of the CS coupons (*n* = 6) were evaluated using a three-electrode system consisting of a UNS G10180 coupon as the working electrode, a graphite rod (Alfa Aesar, 99.9995%, 6.15 mm diameter, 152 mm long) as the counter electrode, and a silver/silver chloride (Ag/AgCl, 3.5 M KCl) reference electrode (Sentek, (AgCl) Double junction Reference Electrode). On day 1, after the test reactor was inoculated, both reactors were left for at least 1 h prior to performing any electrochemical measurements. Open-circuit potentials (OCP) were recorded for each coupon on day 1 prior to measuring linear polarization resistance (LPR) and electrochemical impedance spectroscopy (EIS). LPR and EIS were measured daily for each sample. LPR measurements were performed from ±10 mV with respect to *E*_OCP_ using a scan rate of 0.167 mV s^-1^. EIS measurements were performed at OCP with an applied 10 mV_rms_ sinusoidal potential signal with a frequency range of 10^−2^ to 10^5 ^Hz. Potentiodynamic polarizations were performed at the end of the experiment on day 28 for each coupon from –0.200 mV to +0.200 V using the scan rate of 0.5 mV s^–1^. Standard practices were followed when selecting an equivalent circuit best-fit using the Gamry Echem Analyst software: (*i*) the chi-squared (*χ*^2^) error was suitably minimized (*χ*^2^ ≤ 10^–4^) and (*ii*) the errors associated with each element were ranged between 0% and 5%.

### Confocal laser scanning microscopy and post-image analysis

The distribution of live and dead cells within biofilms was studied using confocal laser scanning microscopy (CLSM). Coupons (*n* = 3) were gently rinsed with sterile anaerobic PBS, with the following composition: ${\rm{NaCl}}$ 8 g, ${\rm{KCl}}$ 0.2 g, ${{\rm{Na}}}_{2}{{\rm{HPO}}}_{4}$ 1.44 g, ${{\rm{KH}}}_{2}{{\rm{PO}}}_{4}$ 0.245 g, deionised water 1 L; and subsequently stained using the FilmTracer Live/Dead biofilm viability kit (Invitrogen) according to the manufacturer’s instructions. Before imaging with a Leica SP8 confocal microscope, coupons were rinsed with sterile deionized water to remove the excess of dyes and fixed using mowiol. Mowiol had the following composition: 2.4 g Mowiol, 6 mL deionized water, 12 mL 0.2 M Tris (pH 8.5), 0.01 g sodium azide, and 6 g glycerol. CLSM images were obtained with a 63× magnification and glycerol immersion. The dyes used stained live cells with a green-fluorescent colour (SYTO 9) and dead cells with a red colour (propidium iodide). The *z*-stacked images were analysed using Imaris software (Oxford Instruments). Live/dead cell ratios were quantified using Imaris (Bitplane) image analysis software by applying intensity-based thresholding and volumetric segmentation to fluorescence microscopy *z*-stacks, enabling the discrimination and enumeration of SYTO 9–stained (live) and propidium iodide–stained (dead) cells within biofilm structures.

### Microbial community analysis

After 28 days, six AR coupons (*n* = 6), were gently rinsed with PBS and then placed in a Falcon tube containing 10 mL of ASW solution. Long-term frozen stocks were prepared using 20% glycerol for the bulk fluid, AR biofilm, and P biofilm samples from the biotic reactor. The sediment, three-day pre-culture, day 28 bulk fluid, AR biofilm, and P biofilm frozen stocks were sent in triplicate for DNA extraction and 16S rRNA amplicon sequencing to Eurofins Genomics LLC. Library preparation and sequencing were performed for the V3 and V4 regions of the 16S rRNA gene, targeting both bacteria and archaea. The microbiome analysis pipeline, along with DNA extractio,n was performed by Eurofins Genomics LLC using a proprietary protocol. The reference for the primer pair used was not disclosed to the authors. The Illumina platform was used, sequencing on MiSeq with the 2 × 300 bp paired-end read module. 2,293,909 raw reads were obtained. Taxonomic classification method using Kraken2 (v 2.1.1). Bioinformatics and data analysis were performed using the Qiime2 (version 2023.5) software. Reference library k2_standard_08gb_20240112 was used for taxonomic classification. To visualize the multivariate dispersion of the community composition, a principal component analysis (PCA) analysis was conducted employing GraphPad (version 10.0.2).

### ATP assay

The ATP concentration in both the abiotic and biotic reactors was determined by luminescence after reaction with luciferin-luciferase using the BacTiter-Glo™ Microbial Cell Viability Assay kit (Promega). The assay provides a method for determining the number of viable microbial cells in culture based on the quantitation of the ATP present. ATP is the energy source of all living cells and is involved in many vital biochemical reactions. When cells die, they stop synthesizing ATP and the existing ATP pool is quickly degraded. Higher ATP concentration indicates a higher number of living cells. All assays were performed according to the manufacturer’s instructions. Six AR coupons were gently rinsed with PBS and then immersed in a Falcon tube containing 10 mL of ASW solution. Any cells were detached from the metal coupons using a cell scraper (Biologix). Both planktonic and sessile samples were processed with the BacTiter-Glo™ Microbial Cell Viability Assay kit, which measures ATP from as few as 10 microbial cells. The ATP concentrations were determined by measuring luminescence with a Clariostar Plus Multimode Microplate Reader (BMG Labtech). Planktonic cells in each reactor were determined following the same method described before; in this case, 10 mL of the bulk test solution was processed with the BacTiter-Glo™ Microbial Cell Viability Assay kit. Negative controls of PBS, deionised water, and ASW solution were used to indicate no ATP activity.

### Corrosion product analysis

Analysis of the corrosion products and biofilms was performed by SEM-EDS and Raman microspectroscopy. Upon retrieval, the coupons were preserved under anoxic conditions in an AnaeroBox with an AnaeroPack (ThermoFisher). For SEM, all images and Energy dispersive X-ray spectroscopy (EDS) measurements were taken using a Hitachi S-3400N II SEM and attached energy-dispersive X-ray spectrometer (Oxford Instruments). Imaging was performed at approximately 15 kV with a working distance of 10 mm at various magnifications (71×, 1000×, and 3000×). EDS analysis used the same parameters and magnifications. For each sample analysed (*n* = 6) areas were scanned at randomly distributed areas over the sample surface (*n* = 15). EDS data was analysed using AZTEC software before being compiled in Microsoft Excel. Raman microspectroscopy experiments were conducted using a Renishaw InVia Raman microscope (Renishaw, UK), with a Leica DM 2500-M bright field microscope and an automated 100 nm-encoded XYZ stage. The samples were excited using a 532 nm laser directed through a Nikon 50× long working distance air objective (NA = 0.5). Raman-scattered signals were separated from the laser illumination at 532 nm using a Rayleigh edge filter, and a diffraction grating (532 nm: 1800 L mm^–1^) dispersed the Raman-scattered light onto a Peltier-cooled CCD (1024 pixels × 256 pixels). Calibration of the Raman shift was carried out using an internal silicon wafer using the peak at 520 cm^–1^. Spectra were acquired over two or three accumulations of between 5 and 20 s each, using laser power of up to 3 mW. Spectra were acquired from a selection of points manually determined using the brightfield imaging mode of the microscope. The spectra obtained were processed using MATLAB (MathWorks).

## Supplementary information

Supplemental Material_GLUT.ver5_Clean-version

## Data Availability

The sequencing data generated in this study have been deposited in the NCBI Sequence Read Archive (SRA) under the accession number PRJNA1238257, related to BioProject accession numbers SRX28056352, SRX28056353, SRX28056354, SRX28056355. Additional data supporting the findings of this study are available from the corresponding author upon reasonable request.
