# Supplementary material for: Evaluating biocide efficacy in mixed-species biofilms: insights from a dual anaerobic biofilm reactor
Source: Npj Mater Degrad. 2025 Jul 31;9(1):97. doi: 10.1038/s41529-025-00628-0 (PMC12313519; doi:10.1038/s41529-025-00628-0)
Supplement: Supplementary file 1 — Supplemental Material_GLUT.ver5_Clean-version [file 41529_2025_628_MOESM1_ESM.pdf]

Evaluating Biocide Efficacy in Mixed-Species Biofilms: Insights from a Dual Anaerobic Biofilm Reactor

Liam Jones[1*], Niall Hanrahan[1,2], Maria Salta[3], Torben Lund Skovhus[4], Kathryn Thomas[5], Timothy Illson[5], Julian Wharton[6*], Jeremy Webb[1,7*]

[1]School of Biological Sciences, University of Southampton, Southampton, United Kingdom

[2]School of Chemistry, Faculty of Engineering and Physical Sciences, University of Southampton, Southampton SO17 1BJ, United Kingdom

[3]Endures, MIC and Biofilm Department, Bevesierweg 1 DC002, 1781 AT Den Helder, The Netherlands

[4]Research Centre for Built Environment, Climate and Water Technology, VIA University College, Horsens, Denmark

[5]DNV, Holywell Park, Ashby Road, Loughborough, United Kingdom

[6]School of Engineering, University of Southampton, Southampton, United Kingdom

[7]National Biofilms Innovation Centre, United Kingdom

***Correspondence:**
Corresponding Author
lmj1n20@soton.ac.uk, j.s.webb@soton.ac.uk, j.a.wharton@soton.ac.uk

Building 85, University of, Southampton SO17 1BJ

Supplementary Material

Abiotic Coupons

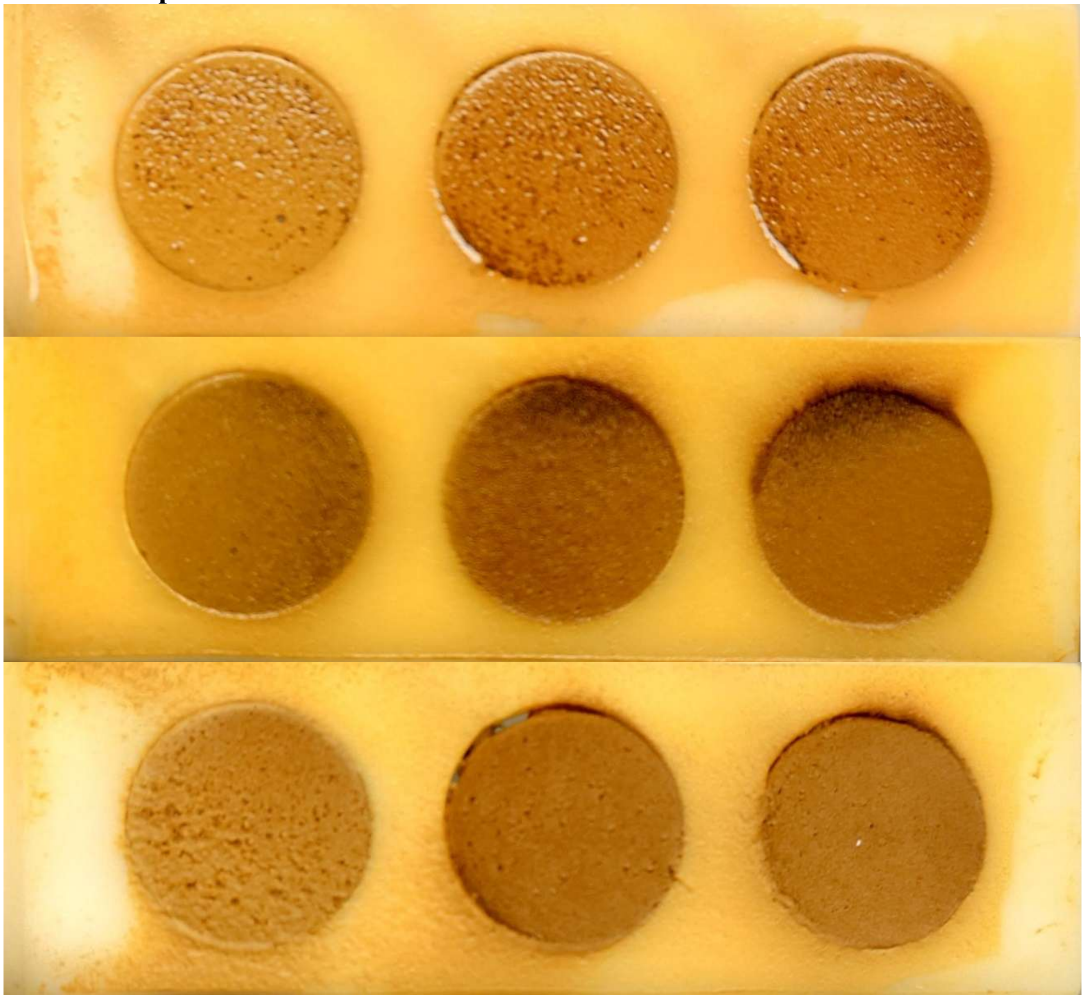

Supplementary Figure 1a. Photographs taken of the coupon rods taken from the abiotic condition on Day 28, on dismantling the reactor, after exposure to anaerobic nutrient-enriched artificial seawater media dosed bi-weekly with glutaraldehyde for 28 days. UNS G10180 (AISI 1018) carbon steel disc coupons, with dimensions of 12.7 mm diameter × 3.8 mm thickness were used as-received (AR).

Biotic Coupons

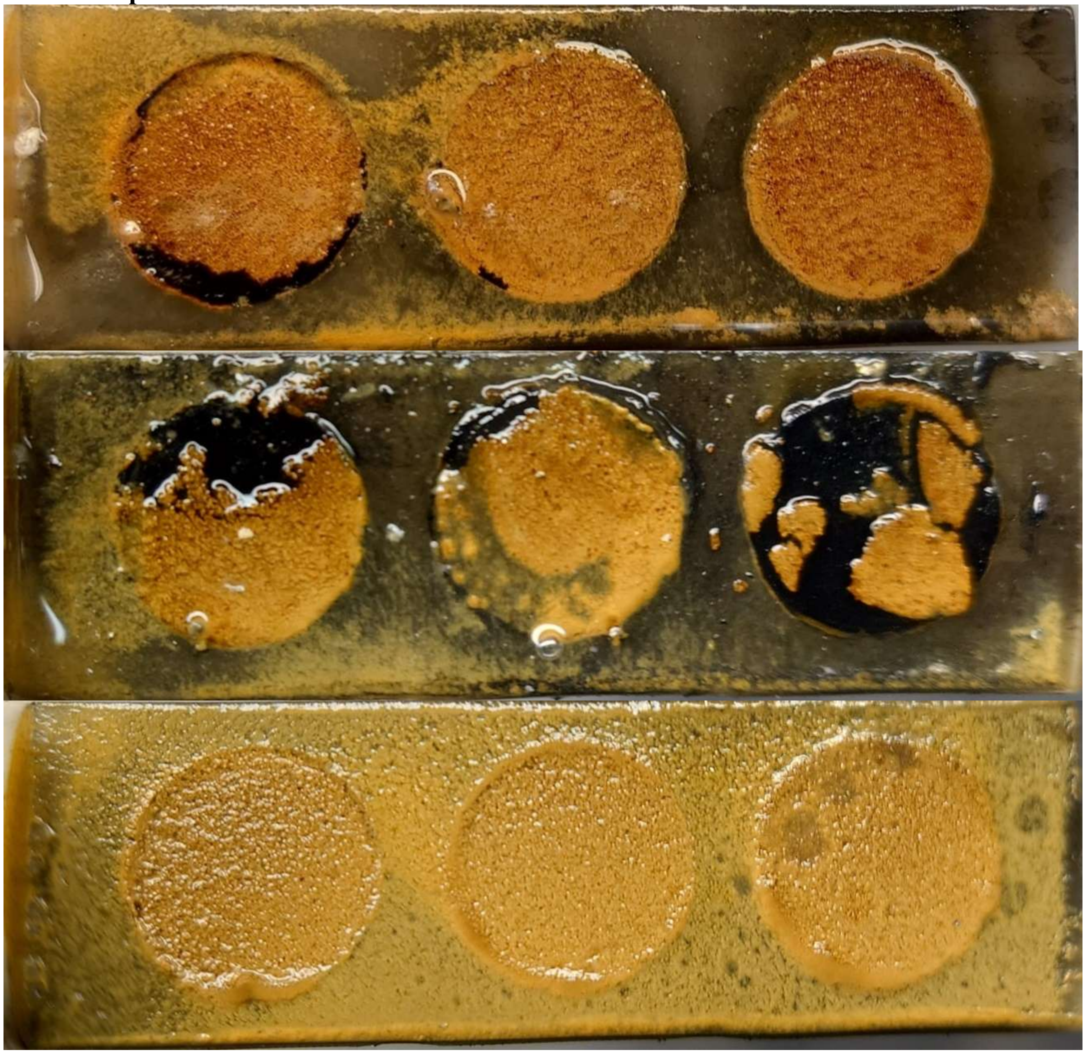

Supplementary Figure 1b. Photographs taken of the coupon rods taken from the biotic condition on Day 28, on dismantling the reactor, after exposure to anaerobic nutrient-enriched artificial seawater media dosed bi-weekly with glutaraldehyde for 28 days. UNS G10180 (AISI 1018) carbon steel disc coupons, with dimensions of 12.7 mm diameter × 3.8 mm thickness were used as-received (AR).

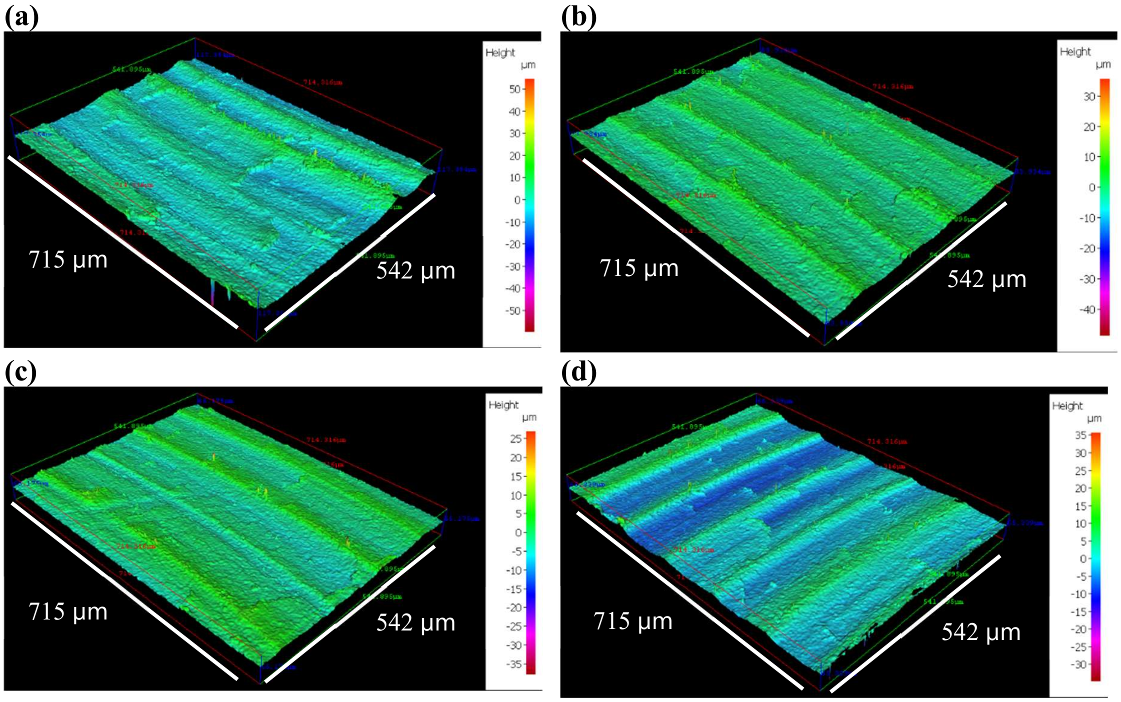

Supplementary Figure 2. Three-dimensional optical surface profilometry of the cleaned UNS G10180 surfaces at day 0. AR coupons for: **(a,b)** abiotic and **(c,d)** biotic conditions, prior to exposure to anaerobic nutrient-enriched artificial seawater media dosed bi-weekly with glutaraldehyde for 28 days.

Supplementary Table 1. Quantitative surface roughness profiles for AR coupon samples on day 0 and day 28. R_a average roughness of profile, R_t maximum peak to valley height of roughness, R_z mean peak to valley height, R_p maximum peak height, R_v maximum valley height, R_c mean height of profile irregularities, R_{sm} mean spacing of profile irregularities, R_t/R_z extreme scratch/peak value of roughness profile (higher values (>=1) represent larger scratches/peaks).

Day	Reactor	R_a (µm)	R_t (µm)	R_z (µm)	R_p (µm)	R_v (µm)	R_c (µm)	R_{sm} (µm)	R_t/R_z (µm)
0	Abiotic	1.2±0.7	9.6±3.7	7.1±2.9	5.7±2.4	3.9±1.7	5.3±2.3	126±34	1.4±0.2
	Biotic	1.3±0.6	10.0±3.2	7.4±2.3	6.3±2.4	3.8±1.1	5.6±2.2	139±38	1.4±0.2
28	Abiotic	1.2±0.6	8.4±3.6	6.6±2.8	4.8±2.2	3.6±1.5	4.7±2.6	104±30	1.3±0.1
	Biotic	1.1±0.5	8.3±2.1	3.4±1.8	4.3±1.2	3.9±1.4	4.4±1.6	104±20	1.3±0.1

Supplementary Table 2a. Quantitative SEM-EDS data collected from elemental mapping of the abiotic UNS G10180 carbon steel surfaces, after exposure to anaerobic nutrient-enriched artificial seawater media dosed bi-weekly with glutaraldehyde for 28 days.

Percentage (%)	O	F	Na	Mg	Si	P	S	Cl	K	Ca	Mn	Fe	Mo	Sn
Max	33.07	0.00	2.49	0.40	0.36	2.86	2.07	3.89	0.00	0.36	1.15	75.49	1.28	1.27
Min	22.56	0.00	0.54	0.40	0.22	0.29	0.32	0.23	0.00	0.27	0.60	61.14	1.28	1.05
Average	25.55	-	1.35	0.40	0.28	1.19	0.72	1.04	-	0.32	0.81	71.08	1.28	1.12
StDev (σ)	2.51	-	0.58	-	0.05	0.64	0.45	1.21	-	0.04	0.22	3.07	-	0.13

Supplementary Table 2b. Quantitative SEM-EDS data collected from elemental mapping of the biotic UNS G10180 carbon steel surfaces, after exposure to anaerobic nutrient-enriched artificial seawater media dosed bi-weekly with glutaraldehyde for 28 days.

Percentage (%)	O	F	Na	Mg	Si	P	S	Cl	K	Ca	Mn	Fe	Mo	Sn
Max	31.62	3.03	5.47	3.20	0.60	2.62	1.92	2.34	0.89	4.25	0.89	73.41	0.00	0.00
Min	21.81	3.03	0.54	0.39	0.20	1.00	0.20	0.19	0.28	0.23	0.51	55.60	0.00	0.00
Average	26.93	3.03	1.89	1.71	0.36	1.61	0.77	0.78	0.52	2.13	0.65	65.05	-	-
StDev	2.37	-	1.56	0.79	0.13	0.48	0.49	0.64	0.26	1.18	0.13	5.20	-	-

Supplementary Figure 3. Equivalent circuit model used to generate EIS parameters shown in Supplementary Table 3.

① R1+(Q1/Q2+R2)

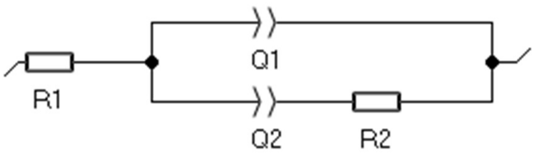

② R1+(Q1+R2)

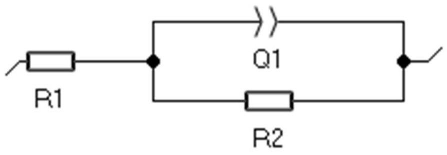

③ R1+Q1/(R2+Q2/R3)

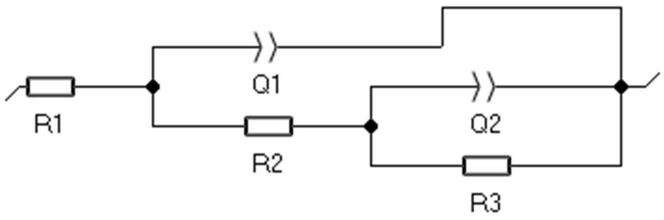

④ R1+Q1+(Q2/R3)

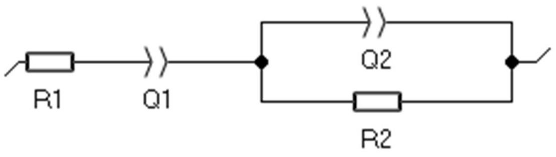

Supplementary Table 3a. EIS parameters of the carbon steel coupons immersed in anaerobic nutrient-enriched artificial seawater media dosed bi-weekly with glutaraldehyde for the abiotic condition on days 1, 7, 14, 21 and 28. **Equivalent circuit:** ① – $R_s+(Q_1/Q_2+R_{ct})$; ② – $R_s+(Q_1/R_{ct})$; ③ – $R_s+Q_1/(R_{film}+Q_2/R_{ct})$.

Day	Coupon	R_s / W cm^2	Q_1 / mW^{-1} cm^{-2} s^n	n_1	R_{film} / W cm^2	Q_2 / mW^{-1} cm^{-2} s^n	n_2	R_{ct} / W cm^2	c^2 ×10^{-4}
1	AR ①	8.95±1.22	0.27±0.03	0.81±0.01	–	1.19±1.32	0.52±0.12	3960±1752	9.2
7	AR ②	8.51±0.87	0.67±0.13	0.90±0.01	–	–	–	5030±1547	6.2
14	AR ②	8.70±0.87	0.95±0.18	0.89±0.01	–	–	–	4859±1276	4.1
21	AR ②	8.60±0.85	1.02±0.20	0.90±0.01	–	–	–	4597±1038	4.5
28	AR ③	9.61±0.96	4.52±3.19	0.48±0.14	33920864±55062319	6.78±3.98	0.69±0.17	55±111	2.5

Supplementary Table 3b. EIS parameters of the carbon steel coupons immersed in anaerobic nutrient-enriched artificial seawater media dosed bi-weekly with glutaraldehyde for the biotic condition on days 1, 7, 14, 21 and 28. **Equivalent circuit:** ① – R_s+(Q_1/Q_2+R_{ct}); ④– R_s+Q_1+(Q_2/R_{ct}).

**Day**	**Coupon**	**R_s / W cm^2**	**Q_1 / mW^{-1} cm^{-2} s^n**	**n_1**	**Q_2 / mW^{-1} cm^{-2} s^n**	**n_2**	**R_{ct} / W cm^2**	**c^2 ×10^{-4}**
1	AR ①	7.68±1.43	167±176	0.30±0.19	3.51±1.02	0.83±0.05	265±57	1.1
7	AR ④	4.94±3.42	106±57	0.58±0.46	21.08±11.01	0.87±0.07	82±67	9.0
14	AR ④	6.55±1.10	104±111	0.67±0.32	65.55±81.51	0.79±0.38	106±98	8.7
21	AR ④	5.92±1.28	464±431	0.28±0.35	124.81±217.40	0.78±0.36	199±130	1.4
28	AR ④	6.65±1.26	40±16	0.84±0.02	543±219	0.12±0.13	273±357	1.1

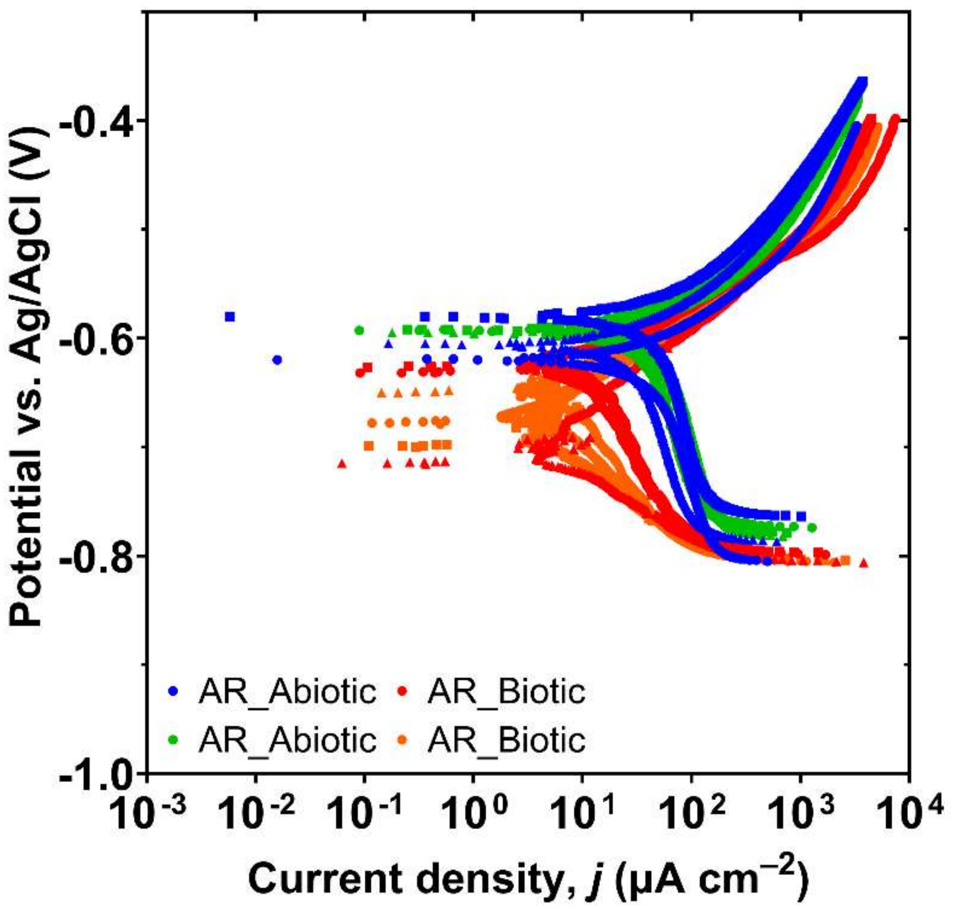

Supplementary Figure 4. Potentiodynamic polarizations for the abiotic and biotic AR, UNS G10180 carbon steel coupons, at ambient temperature after exposure to anoxic nutrient-enriched artificial seawater media dosed bi-weekly with glutaraldehyde for 28 days. Scan rate of 0.5 mV s^{-1} and reactor stirrer at 50 rpm. Performed at the end of the experiment on day 28 for each coupon (*n*=6) from –0.200 mV to +0.200 V using the scan rate of 0.5 mV s-1.

Supplementary Table 4. Fitted electrochemical parameters from polarization curves; comparison between the abiotic and biotic AR, UNS G10180 carbon steel coupons, after exposure anaerobic nutrient-enriched artificial seawater media dosed bi-weekly with glutaraldehyde for 28 days.

	Coupons	j_{corr} / µA cm^{-2}	E_{corr} *vs.* Ag/AgCl / V	β_a (mV dec^{-1})	β_c (mV dec^{-1})
**Abiotic**	AR	0.169 ± 0.035	-599 ± 14	160 ± 6	-
**Biotic**	AR	0.044 ± 0.036	-663 ± 35	113 ± 12	-

Supplementary Table 5. List of top 25 microbial genera identified, as a relative abundance (%), through 16S rRNA amplicon sequencing with two target region, V3-4 for bacteria and archaea, after exposure to anaerobic nutrient-enriched artificial seawater media dosed bi-weekly with glutaraldehyde for 28 days.

Name	Sediment	Day0	Day28	AR
Exiguobacterium	0.003808	0.02348	54.96	70.16
Fusobacterium	0.001632	32.53	0.00535	0.006864
Vibrio	0.08542	6.611	14.94	8.264
Sulfurovum	10.8	0.008712	0.000502	NA
Oceanicoccus	0.02367	10	1.881	2.013
Shewanella	0.02911	0.005631	9.076	2.013
Methanococcoides	0.1387	7.771	0.00117	0.006374
Desulfuromonas	6.802	0.004462	0.000167	NA
Serpentinicella	0.005985	0.6435	2.17	6.596
Desulfosarcina	5.944	0.00425	0.000334	7.00E-05
Thiohalobacter	5.268	0.002762	NA	NA
Photobacterium	0.00272	4.897	0.04163	0.007285
Candidatus Methanoplasma	4.885	0.05716	NA	NA
Blautia	0.006529	4.732	0.0107	0.03754
Candidatus Prometheoarchaeum	4.104	0.02433	NA	7.00E-05
Marinobacterium	0.01387	0.001594	4.001	0.03159
Leptotrichia	0.001904	3.931	0.000836	0.001191
Halarcobacter	0.001088	0.0017	3.578	3.316
Maridesulfovibrio	0.01768	3.475	1.68	0.5737
Pseudodesulfovibrio	3.017	0.1271	0.2426	0.2431
Anaerotignum	0.000816	2.844	0.1206	0.4397
Clostridium	0.2535	2.544	0.03645	0.1919
Urechidicola	2.416	0.000531	NA	NA
Kineobactrum	2.337	0.0017	0.000167	NA
Woeseia	2.26	0.001169	0.000167	NA

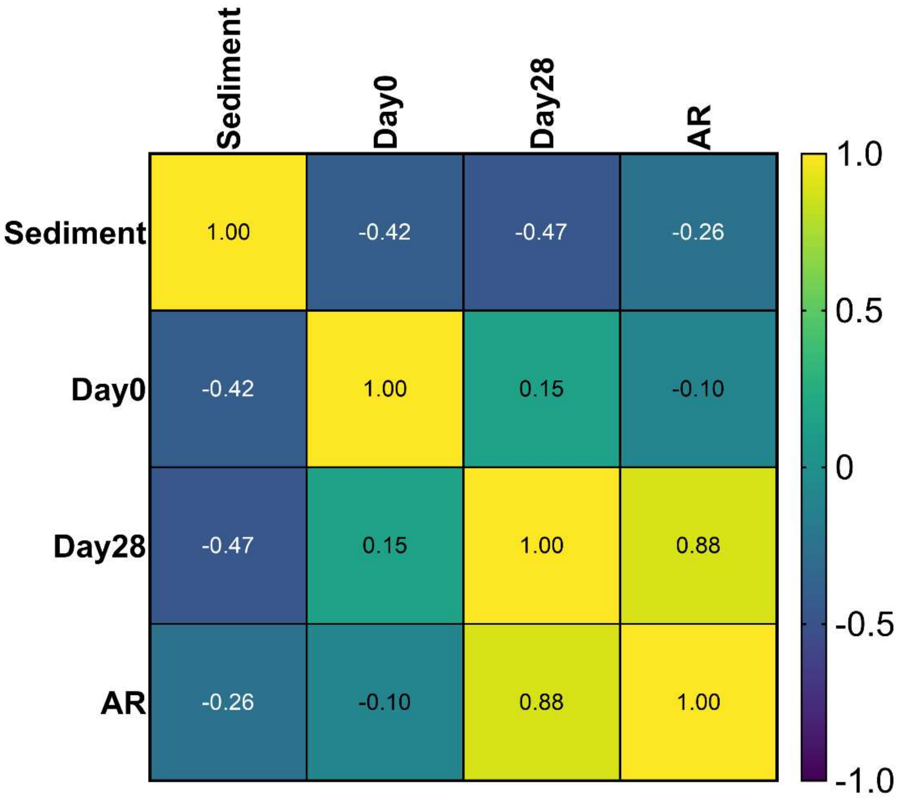

Supplementary Figure 5. Spearman correlation coefficients for environmental marine sediment, Day 0, and Day 28 planktonic samples, and AR biofilms, after exposure to anaerobic nutrient-enriched artificial seawater media dosed bi-weekly with glutaraldehyde for 28 days.

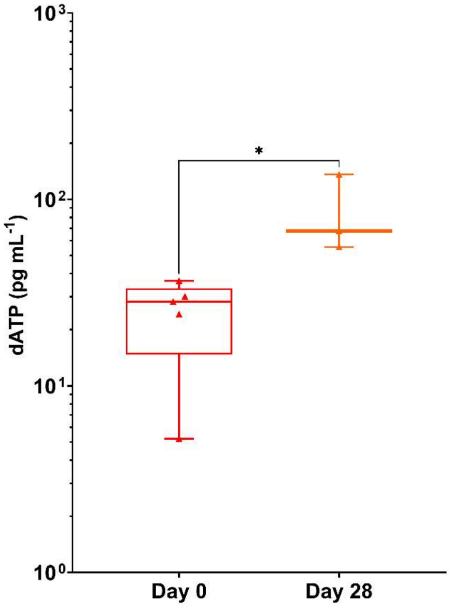

Supplementary Figure 6. Dissolved ATP (dATP) concentrations comparing the anaerobic nutrient-enriched artificial seawater media dosed bi-weekly with glutaraldehyde, taken on Day 0 and Day 28 ($P < 0.05$).

Supplementary Table 6. Artificial seawater test solution/media composition supplemented with 1000 mg L^{-1} of yeast extract. Yeast extract has a protein content of about 50%, of which about 20% is glutathione, 6% is nucleic acid. It is rich in 18 kinds of amino acids, functional peptides glutathione, dextran, mannan, trehalose, flavouring nucleotide, B vitamins, biotin, trace elements and volatile aromatic compounds and other components. Calcium, phosphorus, and trace element contents (μg g^{-1}) in yeast extract: Calcium 1120, Phosphorus 18020, Zinc 190, Iron 162, Chromium 5, Potassium 9300, Cobalt 1.2, Manganese 15, Strontium 3.5, Magnesium 2150.

**Major Ion**	**% Total Weight**	**Concentration (mg L^{-1})**
Chloride, Cl^-	47.47	18,740
Sodium, Na^+	26.28	10,454
Sulfate, SO_4^{2-}	6.6	2,631
Magnesium, Mg^{2+}	3.23	1,256
Calcium, Ca^{2+}	1.013	400
Potassium, K	1.015	401
Bicarbonate, HCO_3^-	0.491	194
Boron, B^{3+}	0.015	6
Strontium, Sr^{2+}	0.001	7.5
Solids Total	86.11%	34.09
Water	13.88	
Total	99.99%	

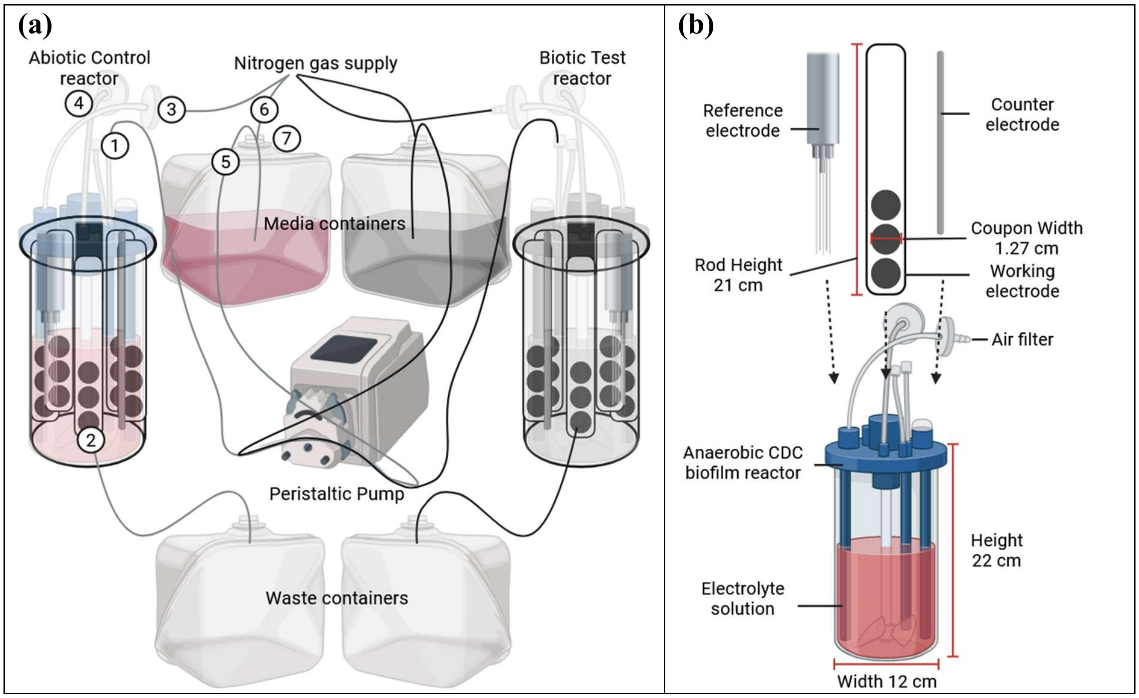

Supplementary Figure 7. (a) The dual anaerobic biofilm reactor system (abiotic and biotic reactors) comprising 10 L media containers, peristaltic pump, magnetic stirrer/hot plate, sulphide microsensor, and the three electrode cell setup. Each reactor has five rods, with three coupons in each rod (15 coupons in total). Each reactor has four inlets. ① The first inlet is connected to the peristaltic pump and then the media container. ② The outlet is connected to the waste containers. ③ Connection to the nitrogen gas source. ④ Air filter (Millex, 0.2 µm) that acts as the exit for excess gas in the reactors. ⑤ The 10 L media container is connected via the peristaltic pump and feeds the first inlet in the reactor. ⑥ Connection to the nitrogen gas source. ⑦ Air filter (Millex, 0.2 µm) that acts as the exit for excess gas in the media containers. **(b)** detailed three-electrode cell setup in an anaerobic CDC biofilm reactor: There are three separate carbon steel coupon working electrodes that can be measured per rod. Each reactor had two rods that were modified for electrochemical analysis, n = 6 for as-received (AR) UNSG10180 carbon steel coupons. Created by BioRender.com.
